# Microwave-Assisted Synthesis and Properties of Novel Hexaazatrinaphthylene Dendritic Scaffolds

**DOI:** 10.3390/molecules25215038

**Published:** 2020-10-30

**Authors:** Daniel García Velázquez, Rafael Luque, Ángel Gutiérrez Ravelo

**Affiliations:** 1 Departamento de Ciencias, Colegio Hispano Inglés, S.A. Rambla de Santa Cruz, 94. 38004 S/C Tenerife, Spain; 2 Departamento de Química Orgánica, Universidad de Cordoba, Campus de Rabanales, Edificio Marie Curie (C-3) Ctra Nnal IV-A, Km. 396 E-14014 Cordoba, Spain; q62alsor@uco.es; 3 Instituto Universitario de Bio-Orgánica “Antonio González”, Universidad de La Laguna, C/Astrofísico Francisco Sánchez, 2, 38206 La Laguna, Tenerife, Spain

**Keywords:** dendrimers, microwaves, hexaazatrinaphthylenes

## Abstract

A novel family of water-soluble π-conjugated hexaazatrinaphthylenes-based dendritic architectures constructed by hexaketocyclohexane and 1,2,4,5-benzenetetramine units is developed in a microwave-assisted organic synthesis (MAOS) approach. The structures and purity of these compounds are verified by ^1^H and ^13^C-NMR, MALDI-TOF MS, UV-vis, elemental analysis, DSC, AFM, STM and cyclic voltammetry.

## 1. Introduction

π-conjugated dendritic architectures have attracted a great deal of attention in recent years as their design and synthesis was shown to render unusual molecular structures and interesting assemblies [1,2]. These dendrimers also possess relevant applications as active chemical components in electronic and optoelectronic devices [2], in biological and material sciences [3], and as photocrosslinkable [4] and photoswitchable devices [5]. Water-compatibility is one of the key properties of such dendritic scaffolds, particularly interesting in view of their utilisation in biological fluids and potentially anti-cancer treatment.

Hexaazatrinaphthylene (HATNA) derivatives are interesting compounds that have a variety of properties [6], including liquid-crystal (discotic) [7], *n*-type semiconduction [8], magnetism [9] and even fluorescence [10], depending on the type of substituent within the structure. Due to this range of properties, an efficient, simple and tuneable preparation of such compounds to make them water-soluble will be highly desirable with regards to their applications and compatibility in aqueous chemistry.

**Figure 1:**
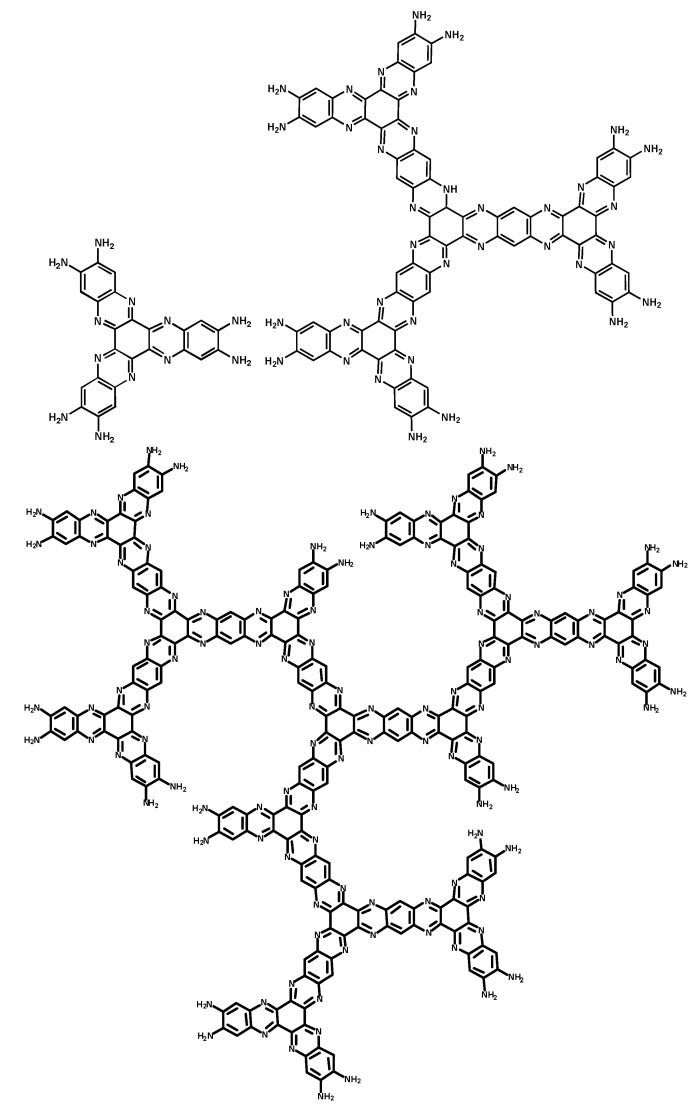
New π-conjugated dendritic architectures of Hexaazatrinaphthylenes (HATNAs) (**G1**, **G2** and **G3**).

With this important concept in mind, herein we report the design and simple preparation of a novel series of water-soluble π-conjugated HATNAs (**G1**, **G2** and **G3**, see Figure 1).

These molecules were synthesized using an efficient microwave-assisted approach from hexaketocyclohexane octahydrate (**1**) and 1,2,4,5-benzenetetramine tetrahydrochloride (**2**) as building blocks. To the best of our knowledge, these compounds are the first examples of dendritic scaffolds based on HATNAs units.

## 2. Results and Discussion

**Figure 2:**
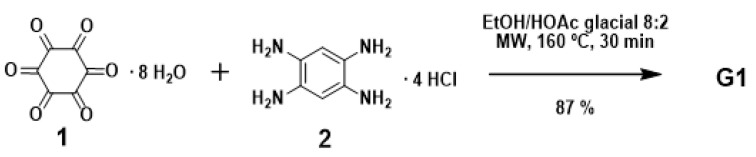
Synthesis of compound **G1**.

**Figure 3:**
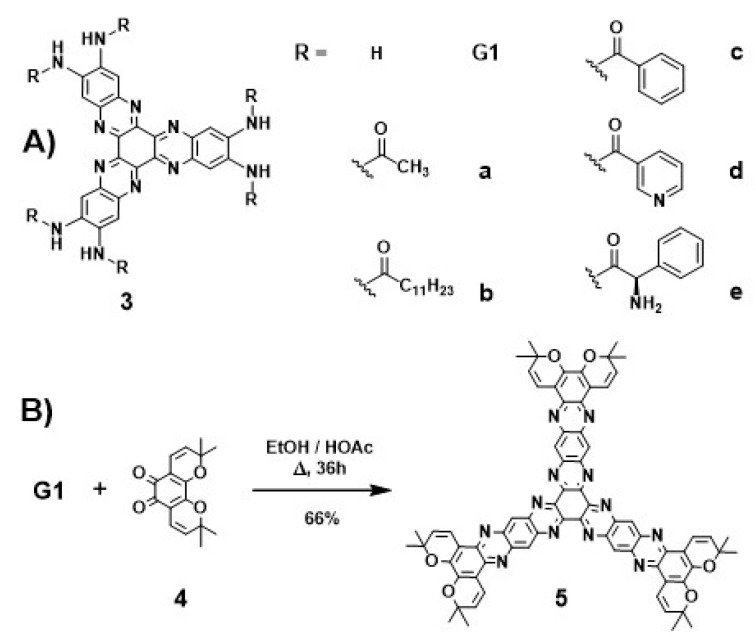
(**A**) Chemical structure of hexaamides **3a**–**e** derived from **G1**. (**B**) Synthesis of **5** for condensation of **4** and **G1**.

The optimized conditions for the synthesis of **G1** were achieved [11] when 1 was heated with 3.75 equiv. of 2 in a mixture of EtOH-HOAc glacial 8:2 under microwave irradiation for 30 min at 160 °C (87% yield) (Figure 2). **G2** and **G3** could be respectively obtained in 82% and 85% yields, under similar reaction conditions (1 equiv. 1 and 3 equiv. **G1** and **G2**, respectively; see ESI). Condensing **G1** and an excess of corresponding acyl chlorides, five derivatives (**3a**–**e**) were synthesized (Figure 3). These types of materials (**3a**–**e**) have six amide groups in the aromatic π-electron system that contribute to the electron-withdrawing effect. Compound **5** was synthesized by condensation of **G1** and orthoquinone **4** [12] as shown in Figure 3.

All compounds were purified by chromatography and crystallization. ^1^H-NMR and ^13^C-NMR spectra, MALDI-TOF MS, UV-vis, FT-IR and elemental analysis, unambiguously proved the structures (see ESI). The self-organization of **G1** into supramolecular nanostructures resulted from the interplay balancing of intramolecular, intermolecular and interfacial interactions. This self-assembly phenomenon was further investigated by ^1^H-NMR, DSC, STM and AFM (see below).

**Figure 4:**
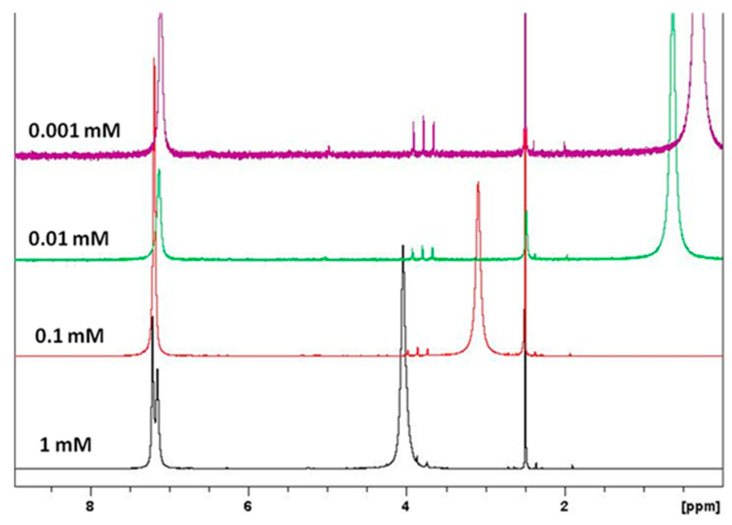
^1^H-NMR spectra of compound **G1** in DMSO-*d*^6^ at 0.001, 0.01, 0.1 and 1 mM at 20 °C.

^1^H-NMR spectra showed that chemical shifts and line widths of **G1** are strongly dependent on the concentration (Figure 4) due to aggregation effects, in good agreement with previous reports.^13^ Molecular interactions are indeed stronger at dilute concentration (*ca*. 10^−5^ M) [13]. ^1^H-NMR chemical shifts (DMSO-*d*_6_) of the aromatic protons for **3a**–**e** and **5** are around δ 6.76–7.83 ppm, moving to higher/lower field as compared with unsubstituted derivate **G1**. The dendritic structures **G1**, **G2** and **G3** present a low solubility in chloroform, dichloromethane and acetone, but are readily soluble in DMF, DMSO, ethanol and water.

**Figure 5:**
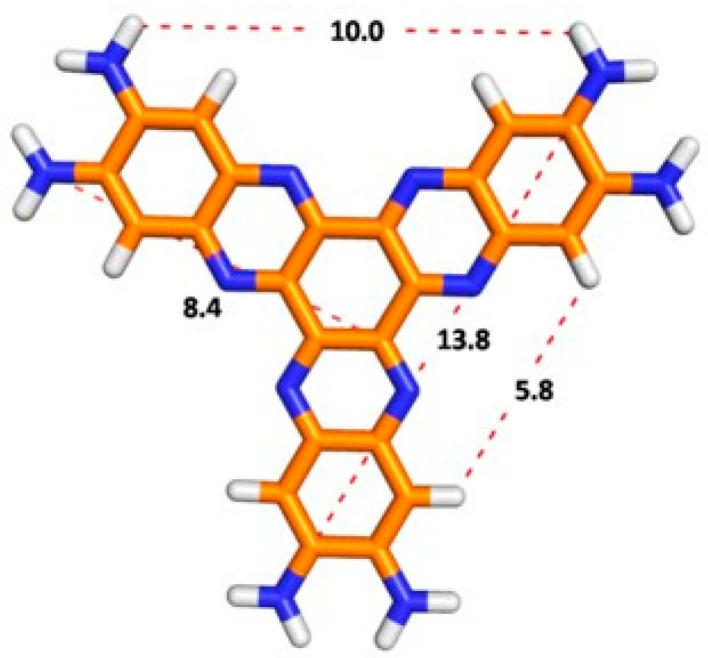
Optimized geometry of **G1** (B3LYP/6-31g*, vacuum) and distances (Å) between atoms.

Therefore, the formation of hydrogen bonds causes the insolubility due to structural defects in columnar ordering that might crosslink neighboring columns via H-bonding, enforcing the intra-columnar stacking order [14]. Thus, neighboring columns crosslinking via hydrogen bonding promote intra-columnar stacking order. Nevertheless, the distortion from the planarity of the aromatic frameworks of **3a**–**e** due to the bulky groups brought high solubilities, presumably through the suppression of aggregation of the aromatic π-systems. Several attempts to crystallize all compounds in different solvent mixtures were unsuccessful, until now. According to molecular modeling, the diameters of **G1** and **G2** are about ca. 16.6 and 29.1 nm (see ESI) with a molecular weight of 474 and 1488 u.m.a., respectively (Figure 5).

**Figure 6:**
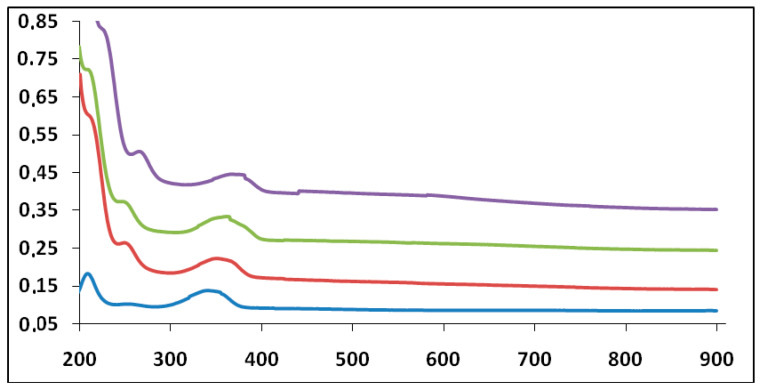
UV-vis spectra (A vs. λ nm) for compound **G1** 10^−5^ M on different days at 20 °C (ethanol as solvent). Day 1 (blue line). Day 3 (red line). Day 5 (green line). Day 7 (violet line) from the preparation of the solution.

When the aggregate of **G1** was formed in a homogeneous aqueous solution at moderate or dilute concentration, the aggregation behavior was analyzed conveniently by spectroscopic methods such as ^1^H-NMR, UV-vis and MALDI-TOF MS (see ESI). The amine groups can maintain a subtle balance between HATNA-HATNA interaction and HATNA-solvent interaction to provide the one-dimensional aggregate, which was confirmed by means of UV-vis spectroscopy (Table 1). In the ethanolic solution, **G1** provides two absorption bands around 209 and 338 nm (Figure 6). The position of the emission maximum peaks undergoes a pronounced bathochromic and hyperchromic effect [15] with an increasing number of days from its preparation (Table 1), which indicates the formation of aggregates. The former two bands can be assigned to the transition from the highest ground state to the ν = 0 level of the lowest excited state (0–0 transition) and to the ν = 1 level (0–1 transition), respectively. The concentration-dependent spectral change was observable in aqueous solutions, which is attributed to dynamic exchange between monomer and aggregate species. Similar photophysical behaviors of three dendritic systems, **G1**, **G2** and **G3**, implied that the effective conjugation length did not improve as the dendritic generation increased.

FT-IR data in the solution state confirm the presence of amino groups and the 1,2,4,5-tetrasubstituted aromatic ring of compounds **G1**, **G2** and **G3**. Theoretically, up to six hydrogen bonds can be formed between successive coplanar disks within the same column. However, the fractions of intra- and inter-molecular hydrogen bonds were not quantified in the present study. FTIR data for **3a**–**e** in the solid-state provides evidence for the existence of hydrogen bonds. The two NH stretching vibrations in IR spectra located at 2910 and 3100 cm^−1^ are shifted to lower energy as compared to that of free NH groups [16]. The presence of only one signal around 1650–1690 cm^−1^ corresponding to the carbonyl group is indicative of the participation of all CO groups in the hydrogen bonds [17].

Table 2 shows the thermal behaviour of **G1**, **G2** and **G3** dendritic assemblies. All compounds possessed high thermal stability and decomposed above 250 °C. Thermal gravimetric analysis (TGA) showed no weight loss up to 275 °C. Glass transition temperatures (*T_g_*) ranged from 142 to 163 °C, while the crystallization transition temperature (*T_c_*) range was 165–238 °C.

**Figure 7:**
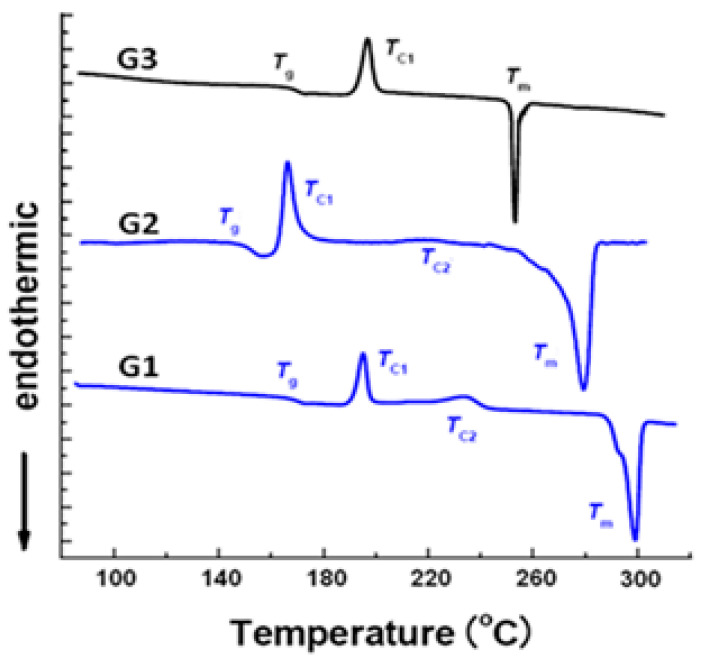
DSC traces of the second heating for compounds **G1**, **G2** and **G3**. All measurements were carried out with heating rate of 10 °C/min.

DSC results showed that **G1**-derivatives **3a**–**e** (HATNA-NHCOR) and **5** did not form columnar liquid crystalline phases as a consequence of the repulsion between adjacent cores (due to the large negatively charged nitrogen atoms) [18]. DSC curves of **3a**–**e** and **5** displayed a broad endothermic peak increasing in intensity from 120 to 270 °C (maximum intensity peak) upon heating from RT to 350 °C (Figure 7).

The associated enthalpy variation (25–76 J·g^−1^) suggests that phase transitions have a strong first-order character. The non-mesogenic behaviour could be related to stabilizing forces induced by van der Waals interactions linked to the aromatic cores charge distribution (see the Mulliken population analysis performed using DFT calculation).

X-ray scattering experiments of **G1** were performed with unoriented powder samples at room temperature (see ESI, Figure S42) and confirmed the columnar mesophase. The X-ray patterns revealed two main features: a series of reflections at relatively small angles and a reflection at large angles corresponding to Bragg spacing of 0.37 nm (core–core separation), indicating a two-dimensional arrangement of the columnar cross-sections in a hexagonal lattice. These data point to the self-organisation of compound **G1** into a columnar π–π stacking phase.

**Figure 8:**
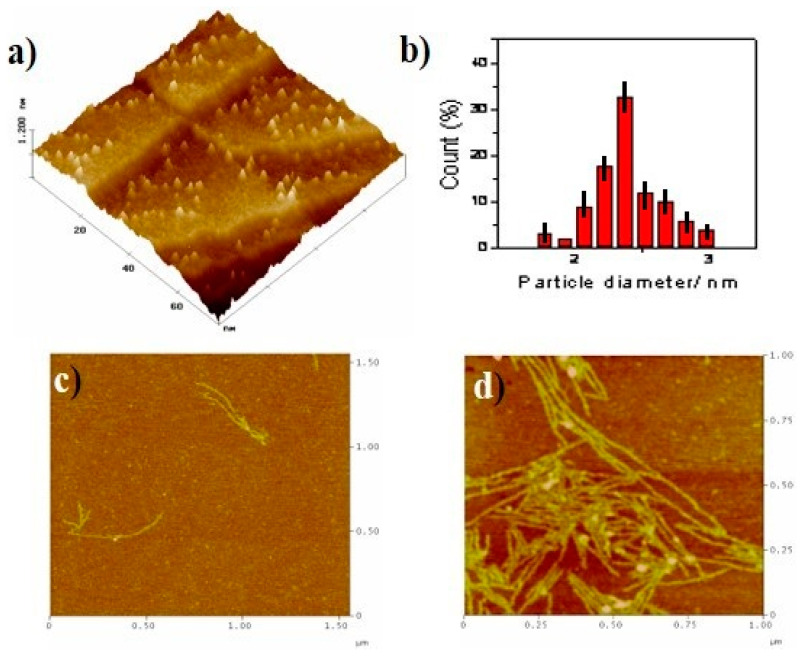
(**a**) 75 nm × 75 nm STM 3D image of the Au(111) surface after 1 min immersion into a **G1** 10^−9^ M water solution. (**b**) Particle size histogram of **G1**. AFM image of mica surface after different times of immersion into a **G1** 10^−5^ M water solution. (**c**) 1.75 µm × 1.75 µm, *t* = 1 min. (**d**) 1.00 µm × 1.00 µm, *t* = 5 min (additional images, see ESI).

STM and AFM experiments were subsequently conducted using different supports, namely Au(111) and mica, in order to confirm the aggregation behaviour of **G1** in aqueous solutions. Isolated discrete particles (less than 200 particles µm^−2^) could be found on the surface of Au(111) as shown in Figure 8a. Although the smallest spots in Figure 8a correspond to particles with sizes in the range of 1.5–3 nm, the majority of them, statistically speaking, are around 2.4 nm (Figure 8b) and range from 2–3 Å width.

Taking into account the molecular dimensions of **G1** (*ca.* 16 Å in size × 2.4 Å high), the simpler units found in aqueous solution should correspond at least to the **G1**-dimer. Comparable results have indeed been observed in related molecules [19].

Comparatively, results obtained for **G1** adsorbed on a mica surface were remarkably different (Figure 8c–d). Two different types of structures grow very fast. Firstly, particles around 12–25 Ǻ in size and 3 Ǻ width appeared randomly distributed on the surface. Secondly, fibers [20] (30–40 nm in size and 4–6 Ǻ width) developed in the material. The number and length of these fibers were increased at longer times of immersion. Therefore, **G1** molecules self-assemble promoting a network of cross-linked fibers in mica. The fact that the width of the fiber is slightly larger than the molecular width would be in good agreement with an “*edge*-*on*” packing of **G1** molecules giving rise to 1D fiber growth, as previously reported in similar disc-like moieties [21].

Interestingly, **G1** molecules seemed to be tilted with respect to a normal surface packing as we can conclude by comparing the diameter of the **G1** molecule (*ca.* 16 Å) with the averaged width of the fibers, (4–6 Ǻ). This is likely to be due to the repulsive interactions between the hydrophobic HATNA cores and the strong hydrophilic mica surface which would in principle restrict a conventional “*lying flat*” position of the molecules. Considering the width of the fibers (30–40 nm), the fibrilar structures most probably comprise of several single stacks in an “*edge*-*on*” arrangement and parallel assembled. These hypotheses may point to a compromise between two main driving forces in the self-assembly of the compounds, namely the π-stacking interfacial interactions (involved in the aromatic cores of **G1** within a single column) and the hydrogen bonding of amine side groups (which promote the intercolumnar packing) [20,21].

In order to ascertain the role played by the π-stacking interactions in **G1** self-assembly, the microscopy study was subsequently extended to the use of highly ordered pyrolytic graphite (HOPG) as a substrate. HOPG has a comparatively larger hydrophobic surface than those of Au(111) and mica.

**Figure 9:**
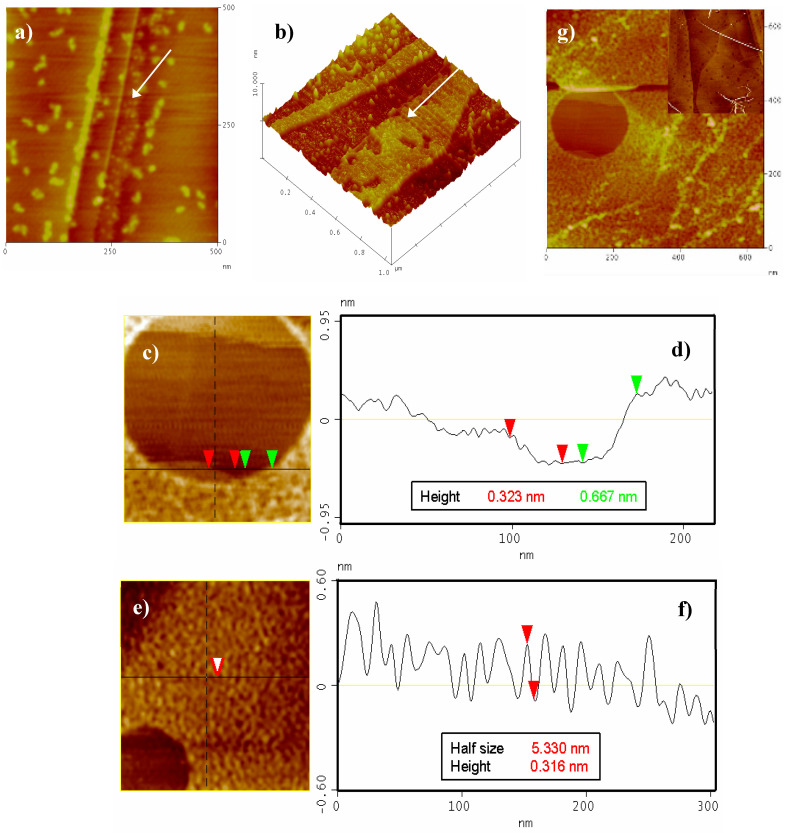
AFM images of pyrolytic graphite (HOPG) after different times of immersion into a **G1** 10^−5^ M water solution. (**a**) 420 nm × 420 nm, *t* = 5 min, (**b**) 1.1 µm × 1.1 µm 3D, *t* = 5 min, (**c**) 220 nm × 220 nm, *t* = 10 min and (**d**) corresponding cross-section shows the first layer (red arrows) and the second layer (green arrows). (**e**) 300 nm × 300 nm, *t* = 10 min and (**f**) cross-section showing the overlayer morphology. (**g**) 620 nm × 620 nm, *t* = 10 min. Inset: 2.8 µm × 2.8 µm, *t* = 5 min, **G1** 10^−4^ M.

Figure 9 shows a monolayer can be clearly seen growing near the HOPG terraces (Figure 9a–b, black arrows). Some big particles can also be found randomly distributed on the clean HOPG terraces. The size of this monolayer (2.6–3.2 Å) is in close agreement with the width of the **G1** molecule lying flat on the HOPG surface, i.e., in a “*face*-*on*” arrangement [21]. Increasing the time of immersion and/or the **G1** concentration leads to an almost complete covering of the HOPG surface by multiple layers resulting from self-assembled molecules (only some void areas left, Figure 9c–g). The majority of the aforementioned voids mostly comprise of the HOPG free surface, a partial **G1** monolayer and a second superposed monolayer (Figure 9c,e,g).

The size of the second layer (*ca*. 6–7 Å, Figure 9d) was twice as great as that observed for the first monolayer, pointing to a π-stacking assembly [22]. No noticeable differences in AFM measurements under phase contrast mode could be observed (not even in the thickness between the first and the second monolayer) [23]. Nevertheless, a different type of packing (molecules in an “*edge-on*” arrangement) cannot be ruled out, especially considering the fiber-like structures shown in Figure 9e,f [21]. Last and most interestingly, new assemblies appear at greater **G1** concentration and/or time of immersion (i.e., long fibers 5–10 nm width and more than one micron long are observed as depicted in Figure 9g and inset). These fibers could only be found on HOPG when the surface was fully covered by several layers of **G1**. Under the investigated conditions, a maximum of three layers was observed. AFM studies on mica and HOPG consequently prove that these layers and fibers grow selectively on the appropriate substrate. Such motives, which constitute a direct consequence of the π-stacking interactions, were not observed on Au(111) (only discrete particles were obtained).

## 3. Experimental Section

*Preparation of 5,6,11,12,17,18-hexaazatrinaphthylene-2,3,8,9,14,15-hexaamine* (**G1**): To a 10 mL reaction vial was added hexaketocyclohexane octahydrate (20 mg, 0,06 mmol, 20 mM) and 1,2,4,5-benzenetetramine tetrahydrochloride (3.75 equiv., 64 mg, 0.22 mmol, 73 mM) followed by 3 mL of 8:2 EtOH-HOAc glacial. The closed vessel was heated and stirred in CEM Discover© reaction cavity for 30 min at 180 °C. Then the reaction vessel was rapidly cooled at 60 °C. Upon cooling, solvents were removed and the black residue was washed with hot glacial acetic acid (3 × 10 mL) and ice water (2 × 10 mL). Drying for 48 h (under vacuum, 5–10 mmHg, 60–80 °C) afforded a violet-black solid as pure product (25 mg, 87%). A sample for analysis was recrystallized from a dichloromethane-ethanol mixture.

*STM* and *AFM* imaging were performed in air with a Nanoscope IIIa microscope from Digital Instruments (Veeco). *Preparation of samples:* Ultrathin dry films of **G1** were prepared from MilliQ water solutions on atomically-flat substrates at room temperature. Samples were prepared by drop casting from diluted water solutions during different times, and then subsequently were thoroughly rinsed with MilliQ water and finally dried during several hours under N_2_ current flow before imaging.

## 4. Conclusions

A simple and efficient synthetic route towards the preparation of HATNA systems was prepared. These peculiar π-conjugated compounds can offer the opportunity to synthesize hierarchically high ordered self-assemblies (e.g., disk-like dendritic supramolecular systems) via π-stacking and the formation columnar anisotropic architectures. The compound **G1** can successfully self-assemble into nanofibers on HOPG and mica surfaces, while only discrete particles were observed on Au(111) surfaces. Optical and electrochemical properties of HATNA compounds as electron-transport materials are currently under investigation in our laboratories.

## Figures and Tables

**Table 1:** UV-vis spectral data for **G1** 10^−5^ M in ethanol at 20 °C.

Days				
**Max. Peak 1**	1	3	5	7
Absorbance	0.1831	0.2611	0.3744	0.6018
λ (nm)	209	244	248	260
ε (M^−1^ cm^−1^)	18,310	26,110	37,440	50,180
Days				
**Max. Peak 2**	1	3	5	7
Absorbance	0.1373	0.2225	0.3326	0.4463
λ (nm)	338	349	355	367
ε (M^−1^ cm^−1^)	13,730	22,250	33,260	44,630

**Table 2:** Mesophase assignment and transition temperatures, °C (onset)*^a^* of dendritic architectures. Glass-transition (*T_g_*), Crystallization (*T_c_*), and Melting (*T_m_*) temperatures of **G1**, **G2** and **G3** compounds (transition enthalpies between parenthesis; J g^−1^).

Compound	*T_g_^a^* [°C]	*T_c1_^a^* [°C]	*T_c2_^a^* [°C]	*T_m_^a^* [°C]
**G1**	163	192 (69)	238 (44)	300
**G2**	142	165 (90)	220 (26)	275
**G3**	160	196 (72)	---	254

*^a^* Measured by DSC at a heating and cooling rate of 10 °C min^−1^. The data from second heating scan and first cooling scan are given and were found to be fully reproducible.

## Supplementary Materials

The following are available on ESI (Electronic Supporting Information).
