# Supplementary material for: Microwave-Assisted Synthesis and Properties of Novel Hexaazatrinaphthylene Dendritic Scaffolds
Source: Molecules. 2020 Oct 30;25(21):5038. doi: 10.3390/molecules25215038 (PMC7663666; doi:10.3390/molecules25215038)
Supplement: Supplementary file 1 [file molecules-25-05038-s001.pdf]

Electronic Supporting Information (ESI)

Microwave-assisted synthesis and properties of novel hexaazatrinaphthylene dendritic scaffolds

Daniel García Velázquez, Rafael Luque and Ángel Gutiérrez Ravelo

Dr. Daniel García Velázquez, Prof. Ángel Gutiérrez Ravelo
Colegio Hispano Inglés, S.A. Departamento de Ciencias. Rambla de Santa Cruz, 94. 38004, S/C Tenerife (España)
E-mail: dgvelazq@ull.es
Dr. Rafael Luque
Departamento de Química Orgánica. Universidad de Córdoba. Campus de Rabanales, Edificio Marie Curie, Ctra Nnal IV_a, Km 396, 14014, Córdoba (España)

- Contents List -

Page
1. General remarks ……………………………………………………........... S2
2. Preparation of compounds……………………………………………........... S4
   2.1. 5,6,11,12,17,18-hexaazatrinaphthylene-2,3,8,9,14,15-hexaamine........... S5
   2.2. 5,6,11,12,17,18-hexaazatrinaphthylene-2,3,8,9,14,15-hexaacetamide.... S5
   2.3. 5,6,11,12,17,18-hexaazatrinaphthylene-2,3,8,9,14,15-hexabenzamide... S6
   2.4. 5,6,11,12,17,18-hexaazatrinaphthylene-2,3,8,9,14,15-hexanicotamide... S7
   2.5. 5,6,11,12,17,18-hexaazatrinaphthylene-2,3,8,9,14,15-hexadodecanamide S7
   2.6. 5,6,11,12,17,18-hexaazatrinaphthylene-2,3,8,9,14,15-hexa(*2R*-amino-*2R*-phenylacetamide)... S8
   2.7. 5,6,11,12,17,18-hexaazatrinaphthylene-2,3,8,9,14,15-hexaaza-tris(2,2,9,9-tetramethyl-2,5,6,9-tetrahydropyrano[3,2-h]chromene)....... S9
   2.8. Tris(5,6,11,12,17,18-hexaazatrinaphthylene) dodecamine…………….. S10
   2.9. Nonakis(5,6,11,12,17,18-hexaazatrinaphthylene) triacontanamine……. S11
3. Spectroscopy characterization of compounds (^{1}HNMR, ^{13}CNMR, UV-vis and MALDI-TOF spectra) ………... S12
4. FT-IR spectra ……………………………………………………….................. S26
5. Cyclic voltammetry……………………….. S27
6. Differential Scanning Calorimmetry (DSC) and Thermal Gravimetric Analysis (TGA)………………….. S28
7. Calculated (B3LYP/6-31G*) 3D structures of compounds……................... S28
8. Mulliken charge distribution……………………………………................... S29
9. Schematic drawing of self-assembled molecules………………................. S31
10. Atomic force and Scanning tunneling micrographs.................................... S32
11. XRPD …………………………………………………………………….. S33
12. References y notes …………………………………………………….. S33

1. General Remarks: Chemicals were purchased and used as received. All air and water sensitive reactions were performed under nitrogen atmosphere. Solvents used were dried by standard distillation procedures or were of p.a. grade and purchased from Aldrich. All reagents were ACS reagent grade or better and were used as received or purified in an appropriate manner. ^{1}H and ^{13}C NMR spectra were obtained on a Bruker Advance 400 spectrometer in DMSO-d_6 and D_2O. Melting points was measured in a Büchi Melting Point B-540 apparatus and are uncorrected. Chromatographic purifications were conducted by column chromatographic using 0.063-0.2 mm silica gel obtained from Fluka. TLC analysis was facilitated by the use of UV light (254 nm) with fluorescent-indicating plates (silica gel on aluminium, Sigma). Elemental analysis was performed on an Elementar Vario EL (Germany) instrument. UV-vis spectra were recorded on on a Perkin-Elmer Lambda 9 spectrophotometer at room temperature. Microwave heating was performed in a commercial single-mode microwave CEM Discover oven. MALDI-TOF MS were conducted on a Bruker BIFLEX III time-of-flight (TOF) mass spectrometer (Bruker Daltonics, Billerica, MA, USA) operated in a positive ion reflection mode at 20 kV accelerating voltage, where the samples were dissolved in DMSO (200 μg/μl) and diluted in water to obtain 0.1% DMSO. In all experiments 0.5 μl of 2,5-Dihydroxybenzoic acid (DHB; 5 mg/ml) was spotted on the MALDI target (600 mm Anchorchip™; Bruker-Daltonics) and air-dried, then 1 μl of sample solution was layered on top of the matrix and air-dried. For one main spectrum, 30 subspectra with 30 shots *per* subspectrum were accumulated. The profiling spectra were calibrated externally with a premix calibration kit (Bruker Daltonic) and the standard mass deviation was less than 10 ppm. FT-IR spectra were obtained on a Bruker IFS 66 instrument with ATR accessories or on a Bruker IFS 28/55 instrument at room temperature the samples were deposited on ATR module, recording the spectrum directly operating at 8 cm^{-1} resolution with 40 scans. Corrections were made for the solvent. Differential Scanning Calorimeter (DSC) data were obtained in a Pyris Diamond DSC (Perkin Elmer) instrument at a scan of 10 ºC min^{-1} following the general protocol where a given amount of compound is placed in a pre-weighted aluminium pan, which was sealed and weighted on a six-decimal plate balance., and after the measurements the pan was weighted again to check for possible leakage. Scanning tunnelling microscopy (STM) and Atomic force microscopy (AFM) imaging were performed in air with a Nanoscope IIIa microscope from Digital Instruments (Veeco). To minimize the thermal drift images were taken only after thermal equilibrium was reached. Commercial Pt-Ir tips and etched silicon tips (TAP150A, 126-169 KHz and 5 N/ m) were used for STM and AFM measurements, respectively. In the case of AFM imaging the tips used because of their low values of spring constant and oscillating frequencies implies a very soft interaction with samples. Atomic resolution on HOPG and the height of monatomic steps on Au(111) were used to calibrate the microscope piezotubes. *Preparation of samples:* Ultrathin dry films of compound were prepared from MilliQ water solutions on atomically-flat substrates at room temperature. Three different substrates have been used, namely: Au (111) (Arrandee™ prepared by flame annealing as elsewhere published), highly ordered pyrolitic graphite (HOPG) and mica (both were purchased from SPI supplies and used freshly cleaved). Samples were prepared by drop casting from diluted water solutions during different times, and then subsequently were thoroughly rinsed with MilliQ water and finally dried during several hours under N_2 current flow before imaging. Film structures were determined by STM operating in the topographic mode at a bias voltage ranging 0.6-1.0 V, tunnelling current of 0.35-0.45 nA, and at scan rates of 2-4 Hz, and by AFM operating in tapping mode at a scan rate of 1 Hz. Voltammetric runs were carried out at room temperature in

a deareated 10^{-3} M G1 *N,N′*-dimethylformamide (DMF) solution. Tetra-*n*-butylammonium perchlorate (TBAP) was used as the supporting electrolyte in a conventional three electrode electrochemical glass cell. In all electrochemical measurements a bare glassy carbon electrode, an Ag/AgCl saturated KCl electrode, and a platinum foil were used as working, reference and counter electrode, respectively. Measurements were performed with an Autolab (Eco Chemie) PGSTAT30 potentiostat-galvanostat at a scan rate of 200 mV s^{-1}. HPLC was performed in a Jasco system (Tokyo, Japan) equipped with a PU-980 pump, a photodiode array detector model MD-2010 Plus, a Rheodyne (California, US) 7725i manual injector and a Supelco Discovery column C-18 reverse phase (250×4.6 mm I.D.), packed with 5 μm particles.

2. Preparation of compounds

Table S1. Reaction conditions assayed to optimize the synthesis of **G1**.

Entry	Equiv. 1	Equiv. 2	Solvents (mL)	Conditions	Energy	T^a (ºC)	Time
**1**	1	3	Pyridine (9)		r.t.	25	6 d
**2**	1	3	MeOH/HOAc (3)	9:1	MW	150	10 min
**3**	1	3	EtOH (3)		MW	150	10 min
**4**	1	3	neat		MW	150	10 min
**5**	1	3	EtOH (3)	Mont. K10	MW	100	10 min
**6**	1	3	Water (3)		MW	95	10 min
**7**	1	3,75	EtOH/HOAc (3)	9:1	Reflux		48 h
**8**	1	3,75	EtOH/HOAc (3)	9:1	MW	160	15 min
**9**	1	3,75	EtOH (3)	Al_2O_3 acid	MW	130	5 min
**10**	1	3,75	Toluene (3)	pyridine	MW	140	5 min
**11**	1	3,75	DMSO/HOAc (3)	9:1	MW	160	10 min
**12**	1	3,75	DMSO (2)		MW	160	10 min
**13**	1	3,75	DMSO (2)		MW	200	8 min
**14**	1	3,75	EtOH/HOAc (3)	9:1	MW	90	30 min
**15**	1	3,75	EtOH/HOAc (3)	9:1	Reflux		3 d
**16**	1	3,75	EtOH/HOAc (3)	9:1	MW	160	30 min
**17**	1	3,75	EtOH/HOAc (3)	8:2	MW	160	30 min
**18**	1	3,75	1,2-dichloroethane (3)		MW	150	15 min
**19**	1	3,75	HOAc (3)		MW	160	15 min
**20**	1	3,75	Ethylenglicol (3)		MW	150	10 min
**21**	1	3,75	Ethylenglicol (3)		MW	150	30 min
**22**	1	3,75	EtOH (3)	$MgSO_4$	MW	160	10 min
**23**	1	3,75	HOAc *glacial* (3)		Reflux		2h
**24**	1	3,75	HOAc *glacial* (3)		MW	160	10 min
**25**	1	3,75	HOAc *glacial* (3)		MW	160	30 min
**26**	1	3,75	Pyridine (9)	N_2	Reflux		3 d
**27**	1	3,75	EtOH (40)	K_2CO_3	Reflux		20 h
**28**	1	3,75	EtOH	HCl 37%	Reflux		24 h
**29**	1	3,75	MeOH	MnO_2	MW	150	15 min
**30**	1	3,75	EtOH/HOAc *glac* (3)	9:1	MW	160	30 min

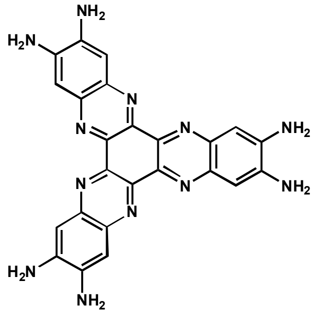

2.1. 5,6,11,12,17,18-hexaazatrinaphthylene-2,3,8,9,14,15-hexaamine (G1):
To a 10 mL reaction vial was added hexaketocyclohexane octahydrate (20 mg, 0,06 mmol, 20 mM) and 1,2,4,5-benzenetetramine tetrahydrochloride (3,75 equiv., 64 mg, 0,22 mmol, 73 mM) followed by 3 mL of 8:2 EtOH-HOAc glacial. The closed vessel was heated and stirred in CEM Discover© reaction cavity for 30 min at 180 ^{0}C. Then the reaction vessel was rapidly cooled at 60 ^{0}C. Upon cooling, solvents were removed and the black residue was washed with hot glacial acetic acid (3 x 10 mL) and ice water (2 x 10 mL). Drying for 48 h (under vacuum, 5-10 mmHg, 60-80 ºC) afforded a violet-black solid as pure product (25 mg, 87%). A sample for analysis was recrystallized from a dichloromethane-ethanol mixture.

Aspect: Violet-dark solid
^{1}H NMR (400 MHz, *d6*-DMSO, δ ppm): 3.10 (12H, s), 7.19 (6H, s)
^{13}C NMR (100 MHz, D_2O, δ ppm): 127.78, 132.79, 138.53, 140.55
m.p.: > 300 ºC (dec.)
FT-IR (EtOH): 810, 825, 885, 1231, 1526, 1559, 1649, 3127 cm^{-1}
UV-vis (c = 10^{-5} M, day 1, H_2O): 209 (log ε 4.26), 338 (4.14)
MS MALDI-TOF (*m/z* %): 474 [M$^+$], 497 [M + Na]$^+$.
Elemental analysis: for $C_{24}H_{18}N_{12}$: 474,18 g/mol. Calcd (%): C: 60.75, H: 3.82, N: 35.42. Found (%): C: 61.28, H: 4.14, N: 35.58.

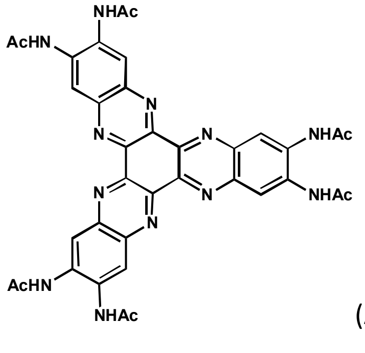

(Ac = acetyl)

2.2 5,6,11,12,17,18-hexaazatrinaphthylene-2,3,8,9,14,15-hexaacetamide (3a):
A flask containing a mixture of **G1** (20 mg, 0,04 mmol), dry pyridine (2 mL) and DMAP (one crystal) in the ice bath at 0 ºC was added acetyl chloride (20 equiv., 0,80 mmol, 61 μL). The solution was warmed to room temperature and was stirred under atmosphere of nitrogen for 12h. Dichloromethane (5 mL) was added, and the organic layer was washed with hydrochloric acid 10% (2 x 5 mL), water (5 mL) and brine (5 mL), and then dried over anhydrous magnesium sulfate, filtered and evaporated to dryness. The residue was dissolved in a mixture of dichloromethane-ethanol (8:2) and filtered through a plug of flash silica and the solvent was concentrated under vacuum

several hours. The residue was recrystallized from ethanol to yield **3a** (28 mg, 96%) as a red-dark solid.

Aspect: Red-dark solid
^{1}H NMR (400 MHz, DMSO-d$_6$, δ ppm): 1.93 (18H, s), 6.85 (6H, s)
^{13}C NMR (100 MHz, DMSO-d$_6$, δ ppm): 27.78, 127.55, 132.48, 139.53, 143.62, 169.99.
Rf TLC plate (UV): 0.25 (*n*-hexane/ethyl acetate 7:3)
m.p.: 204-206 ºC
FT-IR (EtOH): 1015, 1276, 1390, 1507, 1558, 1709, 2617, 2937 cm^{-1}
MS MALDI-TOF (*m/z* %): 726 [M^{+}], 749 [M + Na]$^{+}$.
Elemental analysis: for $C_{36}H_{30}N_{12}O_6$: 726,24 g/mol. Calcd (%): C: 59.50, H: 4.16, N: 23.23. Found (%): C: 59.82, H: 4.14, N: 23.58.

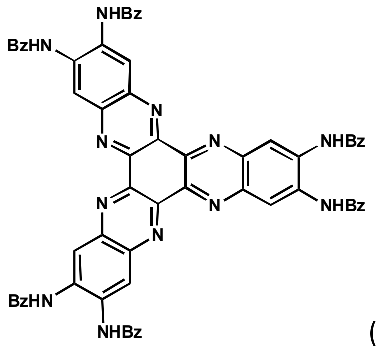

(Bz = benzoyl)

2.3. 5,6,11,12,17,18-hexaazatrinaphthylene-2,3,8,9,14,15-hexabenzamide (3c):
A flask containing a mixture of G1 (20 mg, 0,04 mmol), dry pyridine (2 mL) and DMAP (one crystal) in the ice bath at 0 ºC was added benzoyl chloride (20 equiv., 0,80 mmol, 93 μL). The solution was warmed to room temperature and was stirred under atmosphere of nitrogen for 24h. Dichloromethane (5 mL) was added, and the organic layer was washed with hydrochloric acid 10% (2 x 5 mL), water (5 mL) and brine (5 mL), and then dried over anhydrous magnesium sulfate, filtered and evaporated to dryness. The residue was dissolved in ethyl acetate and filtered through a plug of flash silica and the solvent was concentrated under vacuum several hours. The residue was recrystallized from ethanol to yield **3c** (37 mg, 85%) as a brown-dark solid.

Aspect: Brown-dark solid
^{1}H NMR (400 MHz, DMSO-d$_6$, δ ppm): 6.84 (6H, s), 7.38 (12H, t, J = 8 Hz), 7.51 (6H, t, J = 9 Hz), 7.86 (12H, d, J = 8 Hz)
^{13}C NMR (100 MHz, DMSO-d$_6$, δ ppm): 127.55, 127.76, 129.53, 132.58, 132.62, 135.94, 137.56, 137.69, 172.95
Rf TLC plate (UV): 0.36 (*n*-hexane/ethyl acetate 1:1)
m.p.: 268-270 ºC
FT-IR (EtOH): 743, 1177, 1280, 1319, 1540, 1559, 1699, 1734, 1771, 3050 cm^{-1}
MS MALDI-TOF (*m/z* %): 1098 [M^{+}], 1121 [M + Na]$^{+}$.
Elemental analysis: for $C_{66}H_{42}N_{12}O_6$: 1098,34 g/mol. Calcd (%): C: 72.12, H: 3.85, N: 15.29. Found (%): C: 71.82, H: 4.10, N: 14.95.

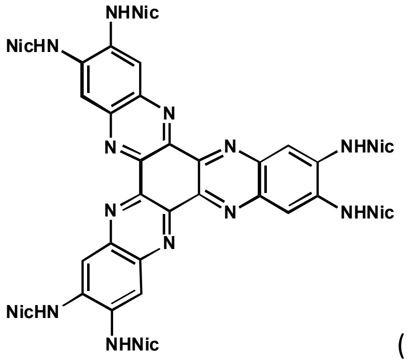

(Nic = nicotinoyl)

2.4. 5,6,11,12,17,18-hexaazatrinaphthylene-2,3,8,9,14,15-hexanicotinamide (3d):
A flask containing a mixture of G1 (20 mg, 0,04 mmol), dry pyridine (2 mL) and DMAP (one crystal) in the ice bath at 0 ºC was added nicotinoyl chloride hydrochloride (20 equiv., 0,80 mmol, 142 mg). The mixture was heated at reflux under atmosphere of nitrogen for 36h. Ethyl acetate (5 mL) was added, and the organic layer was washed with hydrochloric acid 10% (2 x 5 mL), water (5 mL) and brine (5 mL), and then dried over anhydrous magnesium sulfate, filtered and evaporated to dryness. The residue was dissolved in a mixture of dichloromethane-ethanol (9:1) and filtered through a plug of flash silica and the solvent was concentrated under vacuum several hours. The residue was recrystallized from ethanol to yield **3d** (36 mg, 80%) as a orange solid.

Aspect: Orange solid
^{1}H NMR (400 MHz, DMSO-d$_6$, δ ppm): 6.80 (6H, s), 8.11 (6H, t, J = 12 Hz), 8.87 (6H, d, J = 9.75 Hz), 9.00 (6H, d, J = 9.6 Hz), 9.23 (6H, s)
^{13}C NMR (100 MHz, DMSO-d$_6$, δ ppm): 123.71, 127.57, 130.80, 132.20, 135.39, 137.69, 138.58, 147.18, 148.13, 164.82.
Rf TLC plate (UV): 0.47 (*n*-hexane/ethyl acetate 1:1)
m.p.: 250 ºC (dec.)
FT-IR (EtOH): 737, 840, 1300, 1398, 1543, 1562, 1703, 1737, 3135 cm^{-1}
MS MALDI-TOF (*m/z* %): 1104 [M$^+$], 1127 [M + Na]$^+$.
Elemental analysis: for $C_{60}H_{36}N_{18}O_6$: 1104,34 g/mol. Calcd (%): C: 65.21, H: 3.28, N: 22.82. Found (%): C: 65.28, H: 3.20, N: 23.18.

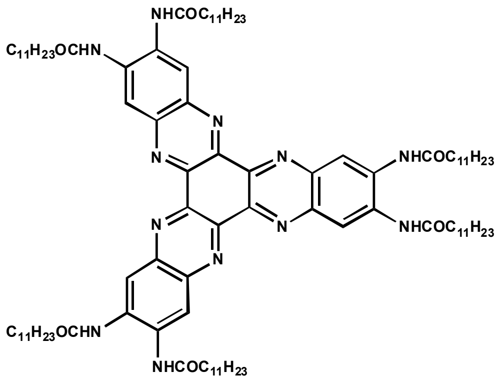

2.5. 5,6,11,12,17,18-hexaazatrinaphthylene-2,3,8,9,14,15-hexadodecanamide (3b):
A flask containing a mixture of G1 (20 mg, 0,04 mmol), dry pyridine (2 mL) and DMAP (one crystal) in the ice bath at 0 ºC was added lauroyl chloride (20 equiv., 0,80 mmol, 185 μL). The reaction was warmed to room temperature and was stirred under atmosphere of nitrogen for 10h. Dichloromethane (5 mL) was added, and the organic

layer was washed with hydrochloric acid 10% (2 x 5 mL), water (5 mL) and brine (5 mL), and then dried over anhydrous magnesium sulfate, filtered and evaporated to dryness. The residue was dissolved in a mixture of dichloromethane-ethanol (8:2) and filtered through a plug of flash silica and the solvent was concentrated under vacuum several hours. The residue was recrystallized from ethanol to yield **3b** (58 mg, 93%) as a blue-grey solid.

Aspect: Blue-grey solid
^{1}H NMR (400 MHz, DMSO-d$_6$, δ ppm): 0.88 (s, 18H), 1.20-1.55 (m, 96H), 2.15-2.33 (m, 12H), 3.01 (t, 12H), 7.05 (s, 6H)
^{13}C NMR (100 MHz, DMSO-d$_6$, δ ppm): 13.94, 22.71, 24.83, 25.06, 28.45, 29.00, 29.24, 29.47, 29.68, 31.84, 32.04, 129.06, 133.54, 140.42, 142.21, 168.80
Rf TLC plate (phosphomolibdic acid): 0.32 (*n*-hexane/ethyl acetate 1:1)
m.p.: 160-163 ºC
FT-IR (EtOH): 711, 735, 800, 863, 1275, 1300, 1539, 1548 1684, 1698, 2848, 2914, 2957 cm^{-1}
MS MALDI-TOF (*m/z* %): 1568 [M$^+$], 1591 [M + Na]$^+$.
Elemental analysis: for $C_{96}H_{150}N_{12}O_6$: 1567,18 g/mol. Calcd (%): C: 73.52, H: 9.64, N: 10.72. Found (%): C: 73.62, H: 9.59, N: 10.76.

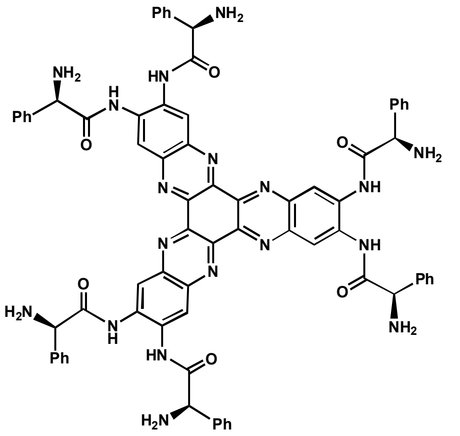

2.6. 5,6,11,12,17,18-hexaazatrinaphthylene-2,3,8,9,14,15-hexa(*2R*-amino-*2R*-phenylacetamide) (3e):

A flask containing a mixture of G1 (20 mg, 0,04 mmol), dry pyridine (2 mL) and DMAP (one crystal) in the ice bath at 0 ºC was added D-(-)-α-phenylglycine chloride hydrochloride (10 equiv., 0,40 mmol, 82 mg). The reaction was warmed to room temperature and was stirred under atmosphere of nitrogen for 48h. Ethyl acetate (5 mL) was added, and the organic layer was washed with hydrochloric acid 10% (2 x 5 mL), water (5 mL) and brine (5 mL), and then dried over anhydrous magnesium sulfate, filtered and evaporated to dryness. The residue was dissolved in a mixture of dichloromethane-ethanol (8:2) and filtered through a plug of flash silica and the solvent was concentrated under vacuum several hours. The residue was recrystallized from ethyl acetate to yield **3e** (45 mg, 89%) as a blue-dark solid.

Aspect: Blue-dark solid
^{1}H NMR (400 MHz, DMSO-d$_6$, δ ppm): 4.94 (6H, s), 6.96 (NH_2, broad signal), 7.40 (12H, t), 7.54 (6H, t), 7.62 (12H, d), 8.98 (12H, s)

^{13}C NMR (100 MHz, DMSO-d$_6$, δ ppm): 57.28, 126.93, 128.19, 130.21, 131.47, 133.75, 136.77, 137.78, 138.79, 167.81
Rf TLC plate (UV): 0.20 (dichloromethane-ethanol 9:1)
m.p.: 220 ºC (dec.)
FT-IR (EtOH): 742, 807, 839, 1157, 1196, 1259, 1542, 1561, 1623, 1654, 1703, 1739, 2962 cm^{-1}
[α]$_D$ = -43 (c = 1, H_2O) (*lit.* D-(-)-α-phenylglycine chloride hydrochloride *commercial*: [α]$_D$ = - 156 (c = 1, HCl)
MS MALDI-TOF (*m/z* %): 1272 [M$^+$], 1295 [M + Na]$^+$.
Elemental analysis: for $C_{72}H_{60}N_{18}O_6$: 1272,49 g/mol. Calcd (%): C: 67.91, H: 4.75, N: 19.80. Found (%): C: 68.06, H: 4.75, N: 19.71.

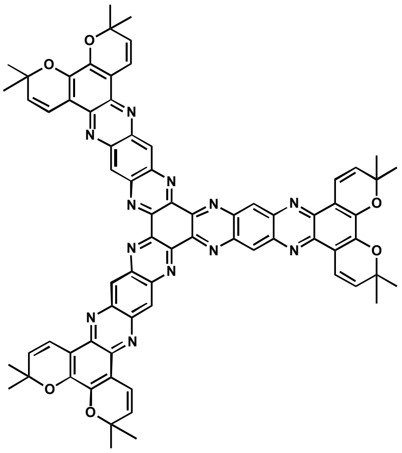

2.7. 5,6,11,12,17,18-hexaazatrinaphthylene-2,3,8,9,14,15-hexaaza-tris(2,2,9,9-tetramethyl-2,5,6,9-tetrahydropyrano[3,2-h]chromene) (5):

To a mixture of G1 (20 mg, 0,04 mmol) and 2,2,9,9-tetramethylpyrano[3,2-h]chromene-5,6(2H,9H)-dione (3.3 equiv., 0,13 mmol, 36 mg) in ethanol (5 mL) was added acetic acid (0.5 mL). The reaction mixture was refluxed under atmosphere of nitrogen until total transformation by TLC (*ca.* 36h). Ethanol was then removed and the residue dissolved in ethyl acetate, followed by washing with bicarbonate solution (2 x 5 mL) and brine (2 x 5 mL) and drying over anhydrous magnesium sulfate, filtered and concentrated under reduced pressure. The crude product was purified by flash silica gel column chromatography (20% EtOAc in *n*-hexane as eluent) to yield **5** (31 mg, 66%) as a red-dark solid.

Aspect: Red-dark solid
^{1}H NMR (400 MHz, DMSO-d$_6$, δ ppm): 1.50 (36H, s), 6.02 (6H, d, J = 6.8 Hz), 6.97 (6H, d, J = 6.5 Hz), 8.14 (6H, s)
^{13}C NMR (100 MHz, DMSO-d$_6$, δ ppm): 28.26, 85.80, 116.59, 117.85, 126.93, 128.19, 129.46, 142.58, 149.90, 151.92
Rf TLC plate (UV): 0.37 (*n*-hexane/ethyl acetate 7:3)
m.p.: 120-122 ºC
FT-IR (EtOH): 737, 1049, 1521, 1538, 1558, 1651, 1683, 1698, 3210 cm^{-1}
MS MALDI-TOF (*m/z* %): 1182 [M$^+$], 1205 [M + Na]$^+$.
Elemental analysis: for $C_{72}H_{54}N_{12}O_6$: 1182,43 g/mol. Calcd (%): C: 73.08, H: 4.60, N: 14.20. Found (%): C: 72.96, H: 4.60, N: 14.13.

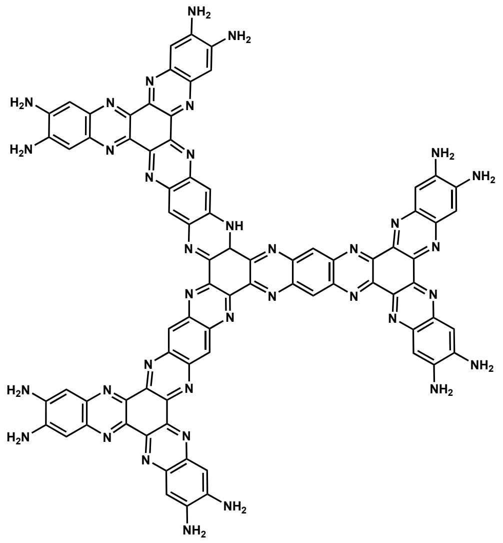

2.8. Tris(5,6,11,12,17,18-hexaazatrinaphthylene) dodecamine (G2):

To a 10 mL reaction vial was added hexaketocyclohexane octahydrate (20 mg, 0,06 mmol, 20 mM) and G1 (3 equiv., 85 mg, 0,18 mmol, 60 mM) followed by 3 mL of 8:2 EtOH-HOAc glacial. The closed vessel was heated and stirred in CEM Discover© reaction cavity for 45 min at 180 ^{0}C. Then the reaction vessel was rapidly cooled at 60 ^{0}C. Upon cooling, solvents were removed and the black residue was washed with hot glacial acetic acid (3 x 10 mL) and ice water (2 x 10 mL). Drying for 48 h (under vacuum, 5-10 mmHg, 60-80 ºC) afforded a green-dark solid as pure product (73 mg, 82%). A sample for analysis was recrystallized from a dichloromethane-ethanol mixture.

Aspect: Green-dark solid
^{1}H NMR (400 MHz, D_2O, δ ppm): 6.82 (1H, s), 7.43 (1H, m, $w_{1/2}$ = 35.29 Hz), 7.83 (1H, m, $w_{1/2}$ = 35.20 Hz)
^{13}C NMR (100 MHz, D_2O, δ ppm): 125.14, 126.64, 128.15, 129.65, 131.75, 135.66, 136.87, 139.57, 142.58, 144.99
m.p.: > 300 ºC (dec.)
FT-IR (EtOH): 793, 817, 840, 880, 1240, 1534, 1600, 1729, 3098 cm^{-1}
MS MALDI-TOF (*m/z* %): 1488 [M^+], 1511 $[M + Na]^+$.
Elemental analysis: for $C_{78}H_{48}N_{36}$: 1488,49 g/mol. Calcd (%): C: 62.90, H: 3.25, N: 33.85. Found (%): C: 62.82, H: 3.14, N: 33.58.

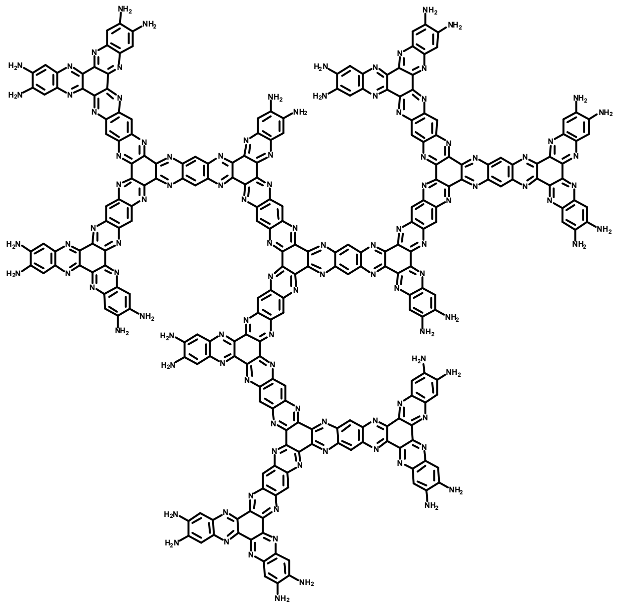

2.9. Nonakis(5,6,11,12,17,18-hexaazatrinaphthylene) triacontanamine (G3):
To a 10 mL reaction vial was added hexaketocyclohexane octahydrate (10 mg, 0,03 mmol, 5 mM) and G2 (3 equiv., 134 mg, 0,09 mmol, 15 mM) followed by 6 mL of 8:2 EtOH-HOAc glacial. The closed vessel was heated and stirred in CEM Discover© reaction cavity for 45 min at 180 ^{0}C. Then the reaction vessel was rapidly cooled at 60 ^{0}C. Upon cooling, solvents were removed and the black residue was washed with hot glacial acetic acid (3 x 10 mL) and ice water (2 x 10 mL). Drying for 48 h (under vacuum, 5-10 mmHg, 60-80 ºC) afforded a red-dark solid as pure product (115 mg, 85%). A sample for analysis was recrystallized from a dichloromethane-ethanol mixture.

Aspect: Red-dark solid
^{1}H NMR (400 MHz, D_2O, δ ppm): 6.76 (12H, s), 6.78 (12H, s), 7.53 (12H, d, J = 1.96 Hz), 7.55 (12H, d, J = 1.95 Hz), 7.57 (12H, d, J = 1.92 Hz)
^{13}C NMR (100 MHz, D_2O, δ ppm): 126.85, 129.07, 129.55, 135.52, 137.44, 137.92, 145.14
m.p.: > 300 ºC
FT-IR (EtOH): 739, 791, 820, 867, 1238, 1532, 1640, 2980 cm^{-1}
MS MALDI-TOF (*m/z* %): 4507 [M^{+}], 4530 [M + Na]$^{+}$.
Elemental analysis: for $C_{240}H_{114}N_{108}$: 4510,22 g/mol. Calcd (%): C: 63.91, H: 2.55, N: 33.54. Found (%): C: 63.96, H: 2.46, N: 33.83.

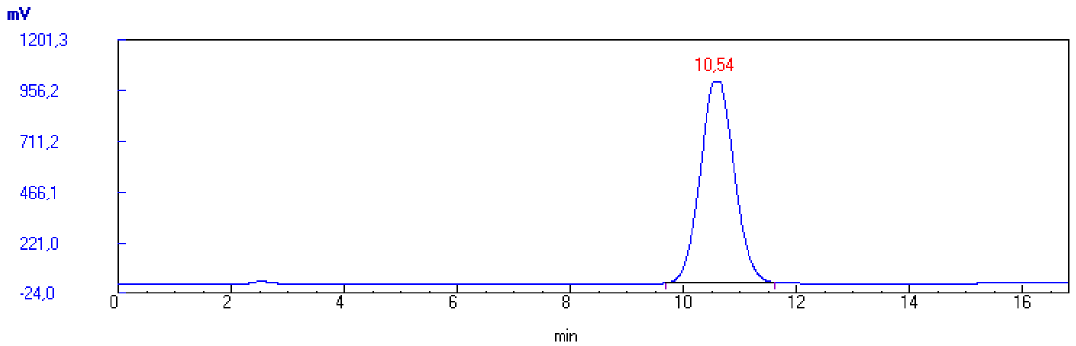

Note: In order to corroborate the presence of only one regioisomer resulting from the reaction, a sample was analyzed by HPLC (mobile phase was a mixture of 50:50 (v/v)

CH_3CN/H_2O, eluted isocratically at a flow rate of 1 mL/min at room temperature and detection conducted at 254 nm.) obtaining a retention time 10.54 min.

3. Spectroscopy characterization of compounds (^{1}H NMR, ^{13}C NMR, MALDI-TOF and UV-vis spectra)

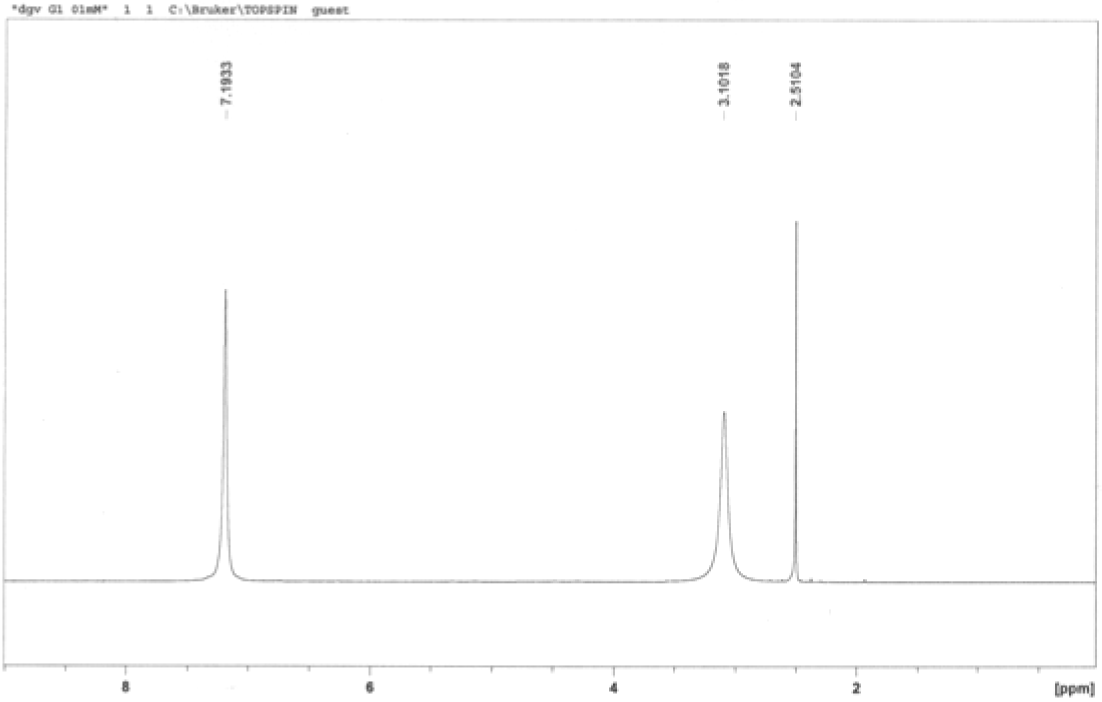

Figure S1. ^{1}H NMR for 5,6,11,12,17,18-hexaazatrinaphthylene-2,3,8,9,14,15-hexaamine (**G1**) in DMSO-d_6

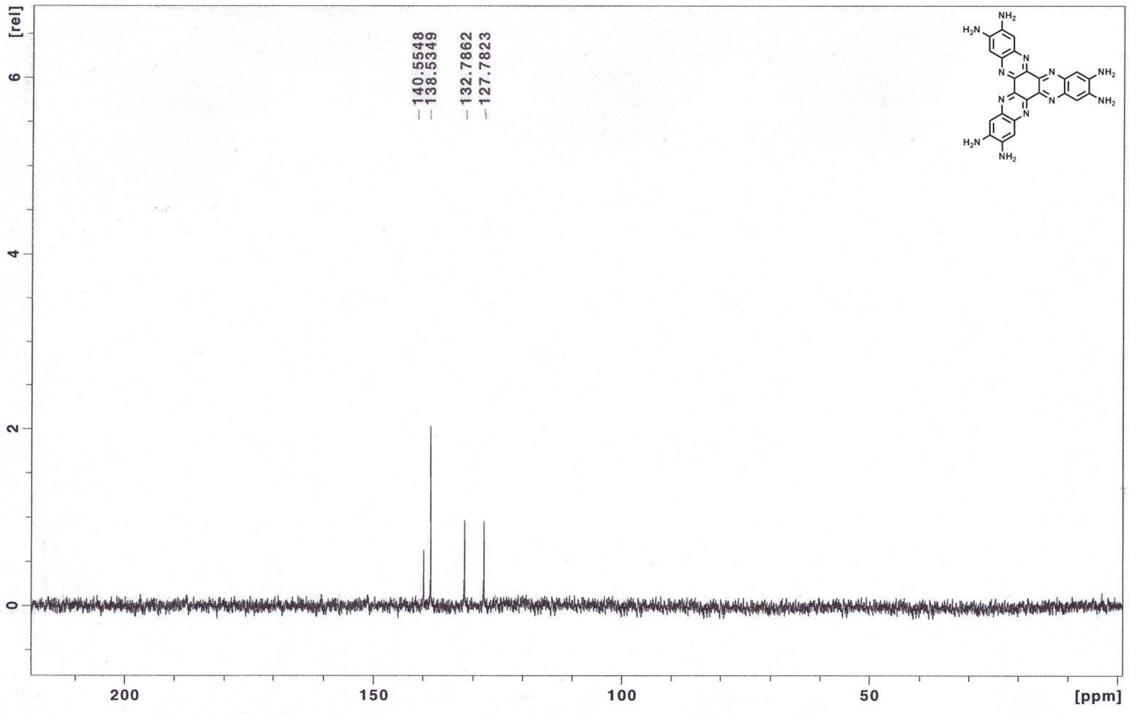

Figure S2. ^{13}C NMR for 5,6,11,12,17,18-hexaazatrinaphthylene-2,3,8,9,14,15-hexaamine (**G1**) in D_2O

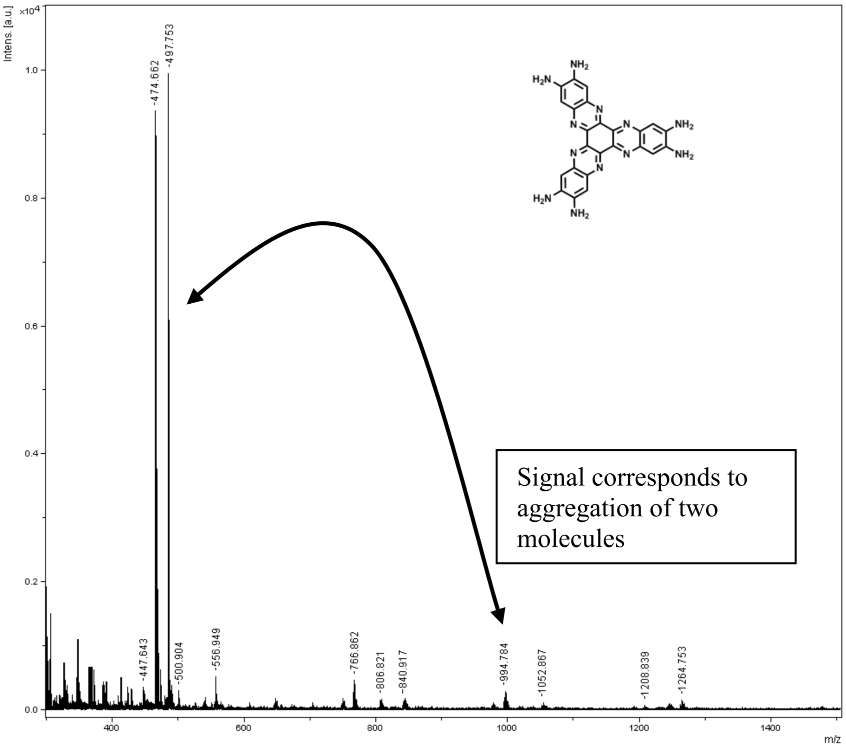

Figure S3. MALDI-TOF for 5,6,11,12,17,18-hexaazatrinaphthylene-2,3,8,9,14,15-hexaamine (**G1**)

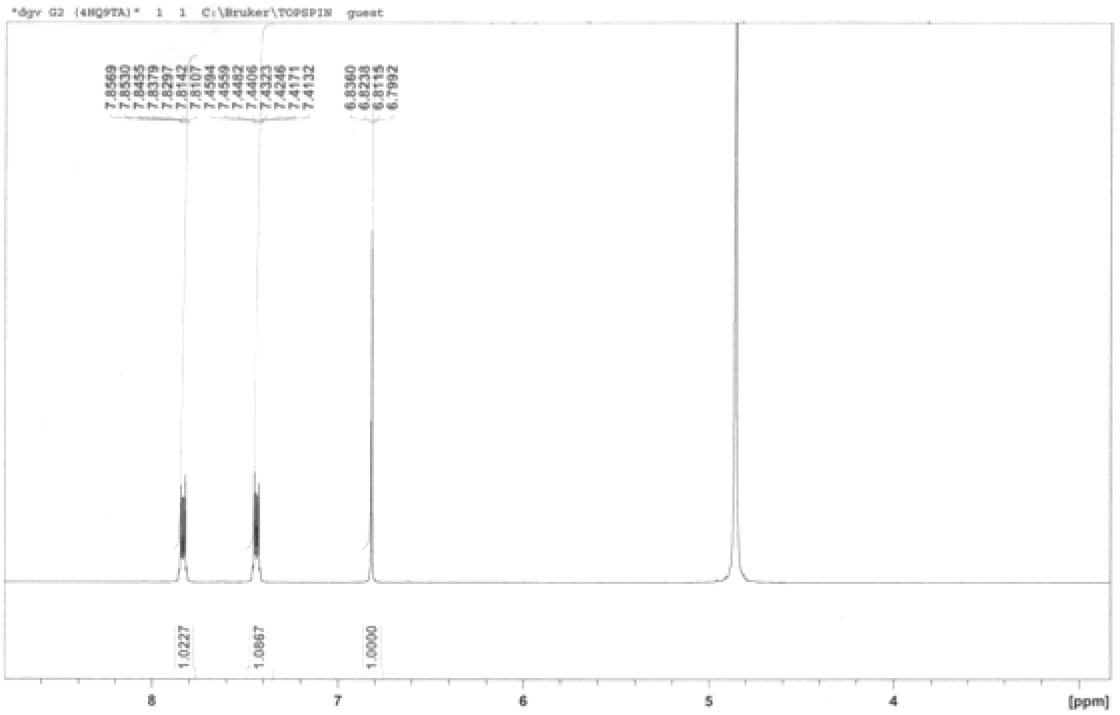

Figure S4. ^{1}H NMR for Tris(5,6,11,12,17,18-hexaazatrinaphthylene) dodecamine (**G2**) in D_2O

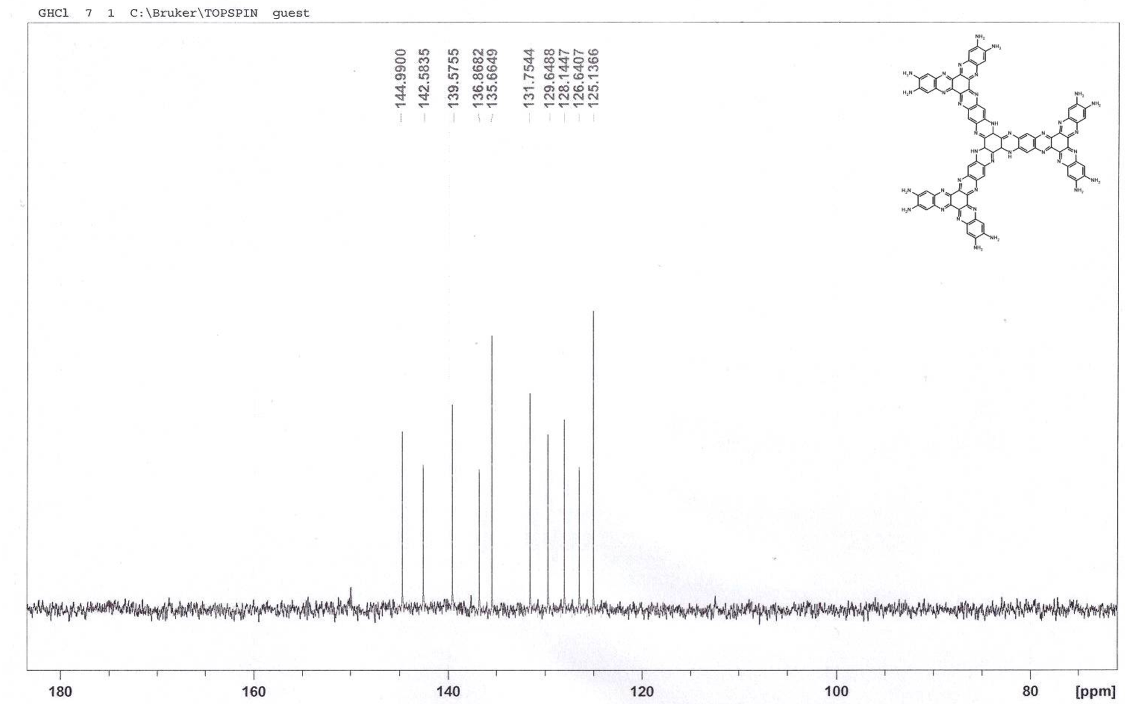

Figure S5. ^{13}C NMR for Tris(5,6,11,12,17,18-hexaazatrinaphthylene) dodecamine (**G2**) in D_2O

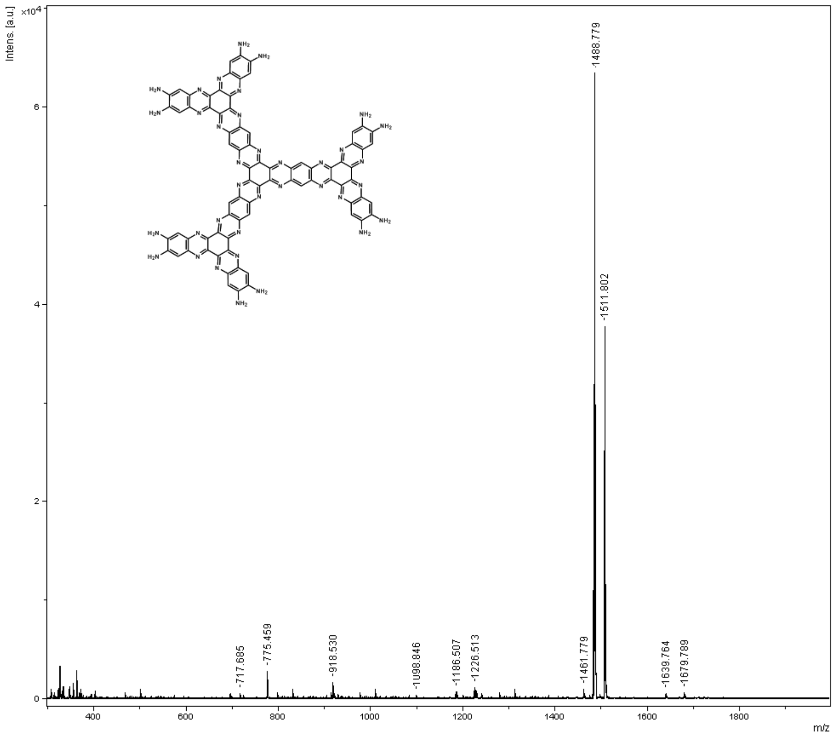

Figure S6. MALDI-TOF for Tris(5,6,11,12,17,18-hexaazatrinaphthylene) dodecamine (**G2**)

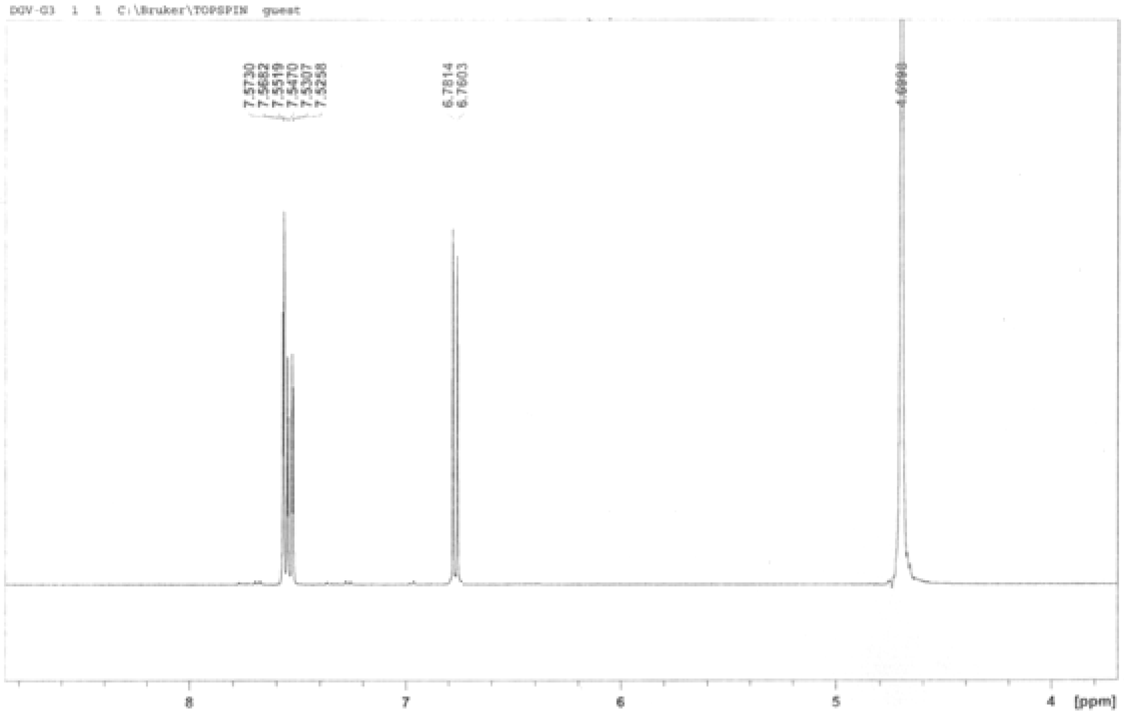

Figure S7. ^{1}H NMR for Nonakis(5,6,11,12,17,18-hexaazatrinaphthylene) triacontanamine (**G3**) in D_2O

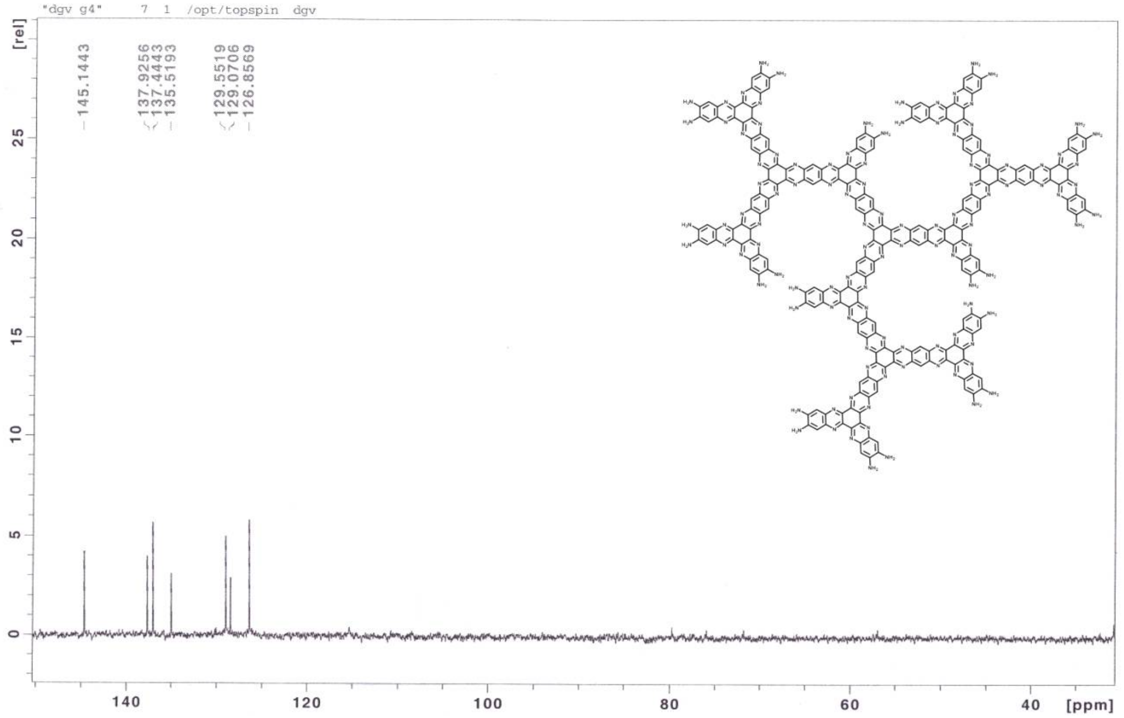

Figure S8. ^{13}C NMR for Nonakis(5,6,11,12,17,18-hexaazatrinaphthylene) triacontanamine (**G´3**) in D_2O

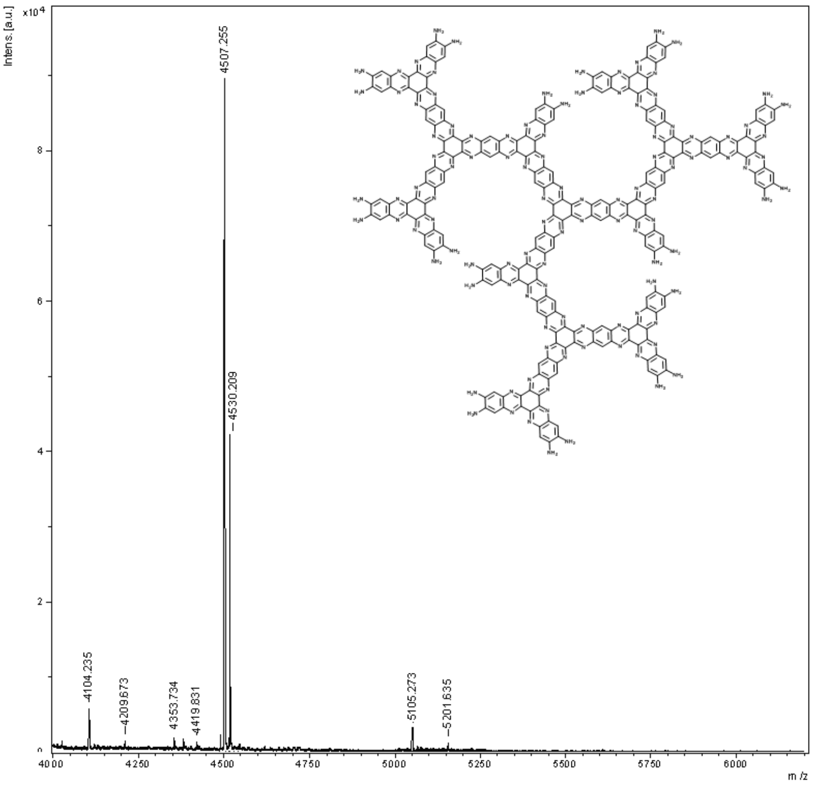

Figure S9. MALDI-TOF for Nonakis(5,6,11,12,17,18-hexaazatrinaphthylene) triacontanamine (**G3**)

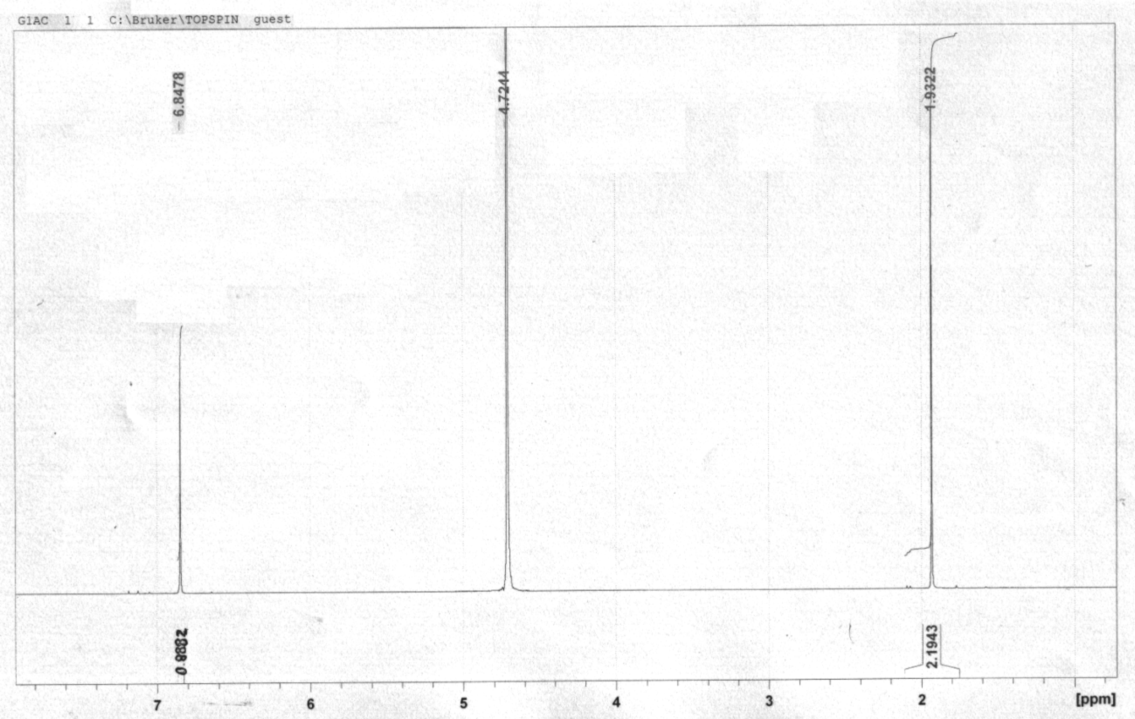

Figure S10. ^{1}H NMR for 5,6,11,12,17,18-hexaazatrinaphthylene-2,3,8,9,14,15-hexaacetamide (**3a**) in DMSO-d_6

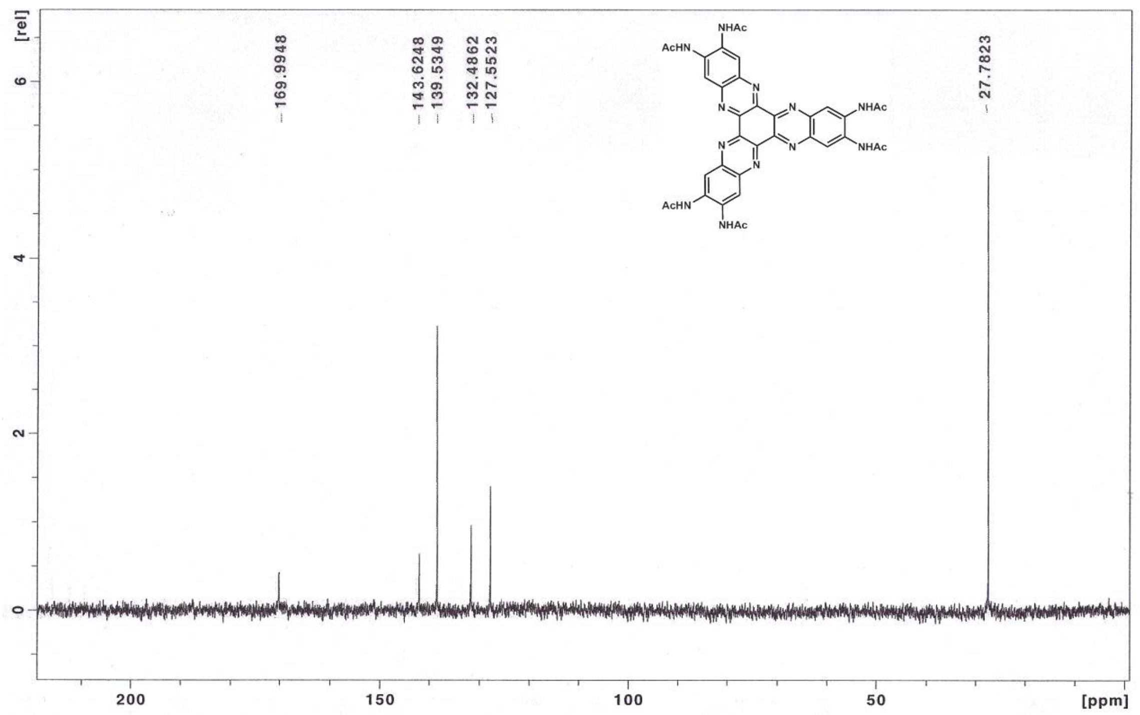

Figure S11. ^{13}C NMR for 5,6,11,12,17,18-hexaazatrinaphthylene-2,3,8,9,14,15-hexaacetamide (**3a**) in DMSO-d_6

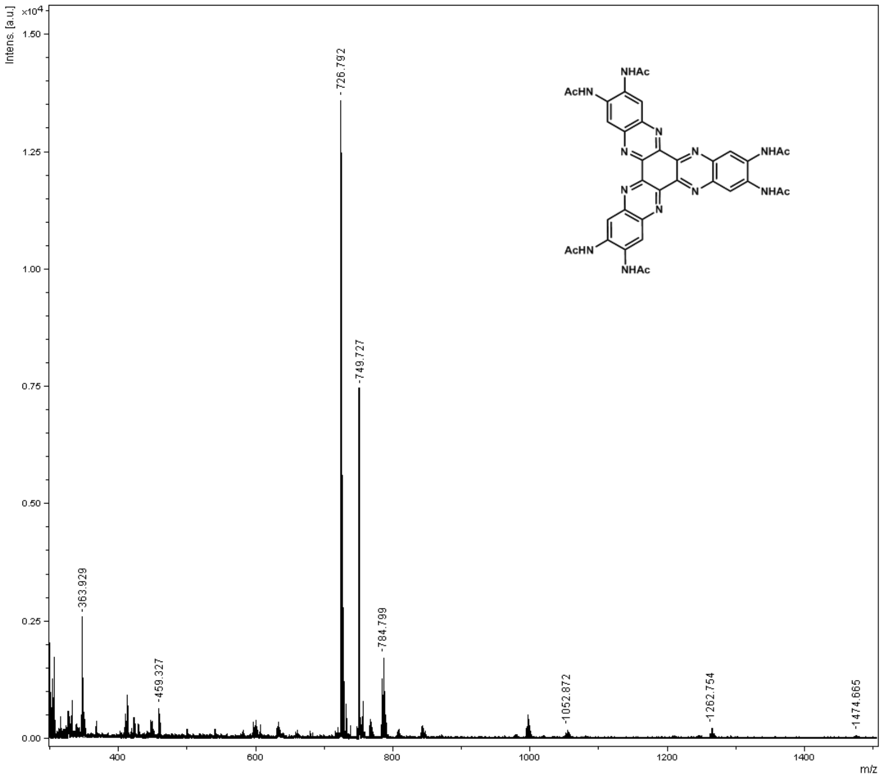

Figure S12. MALDI-TOF for 5,6,11,12,17,18-hexaazatrinaphthylene-2,3,8,9,14,15-hexaacetamide (**3a**)

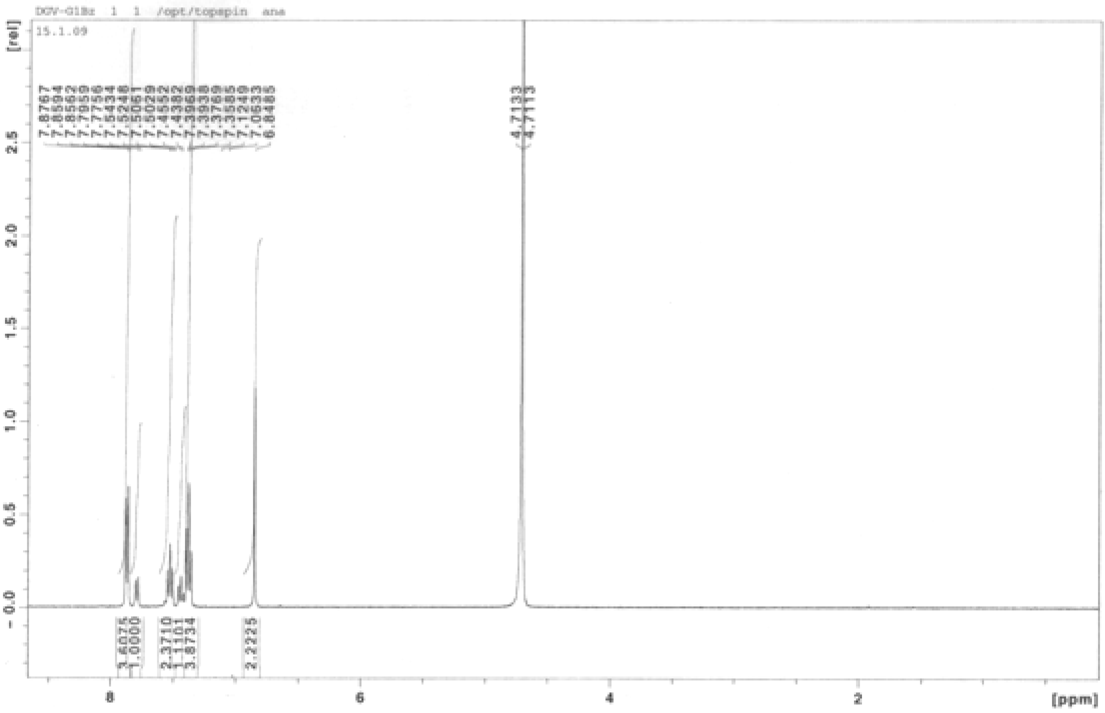

Figure S13. ^{1}H NMR for 5,6,11,12,17,18-hexaazatrinaphthylene-2,3,8,9,14,15-hexabenzamide (**3c**) in DMSO-d_6

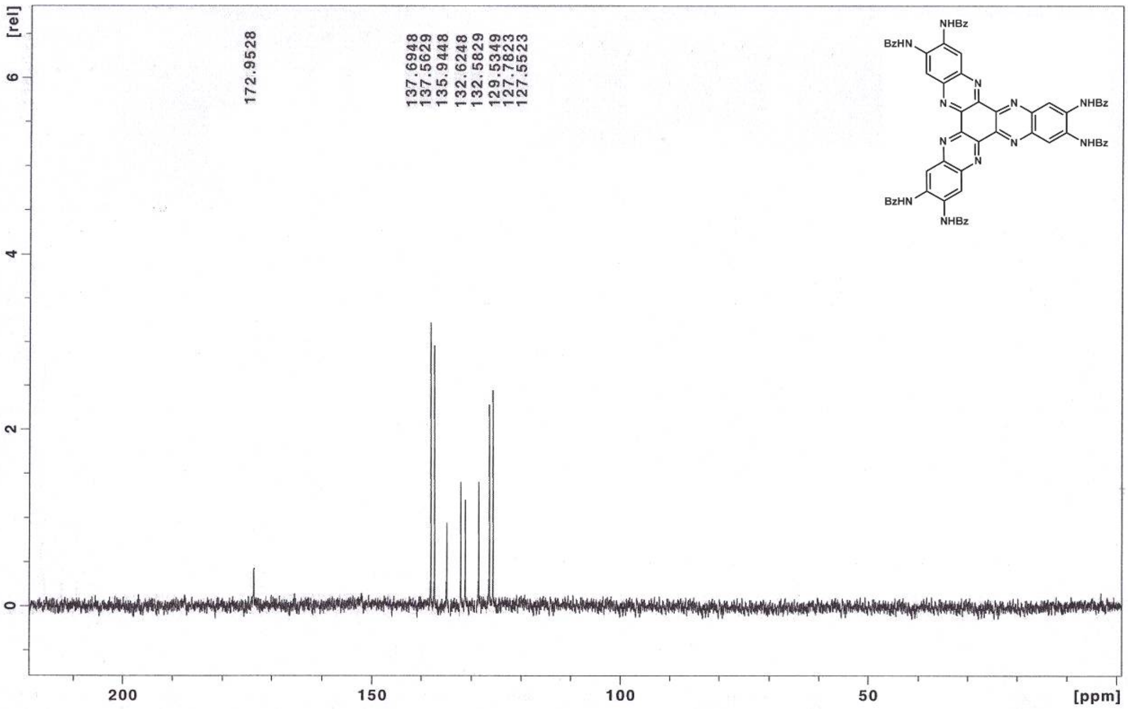

Figure S14. ^{13}C NMR for 5,6,11,12,17,18-hexaazatrinaphthylene-2,3,8,9,14,15-hexabenzamide (**3c**) in DMSO-d_6

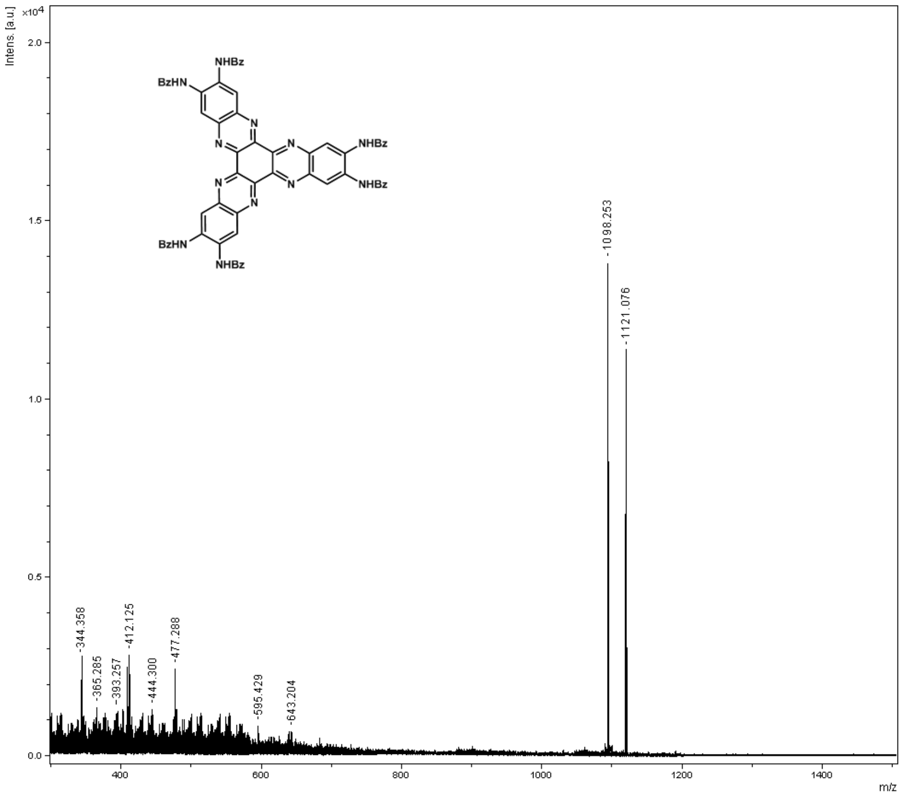

Figure S15. MALDI-TOF for 5,6,11,12,17,18-hexaazatrinaphthylene-2,3,8,9,14,15-hexabenzamide (**3c**)

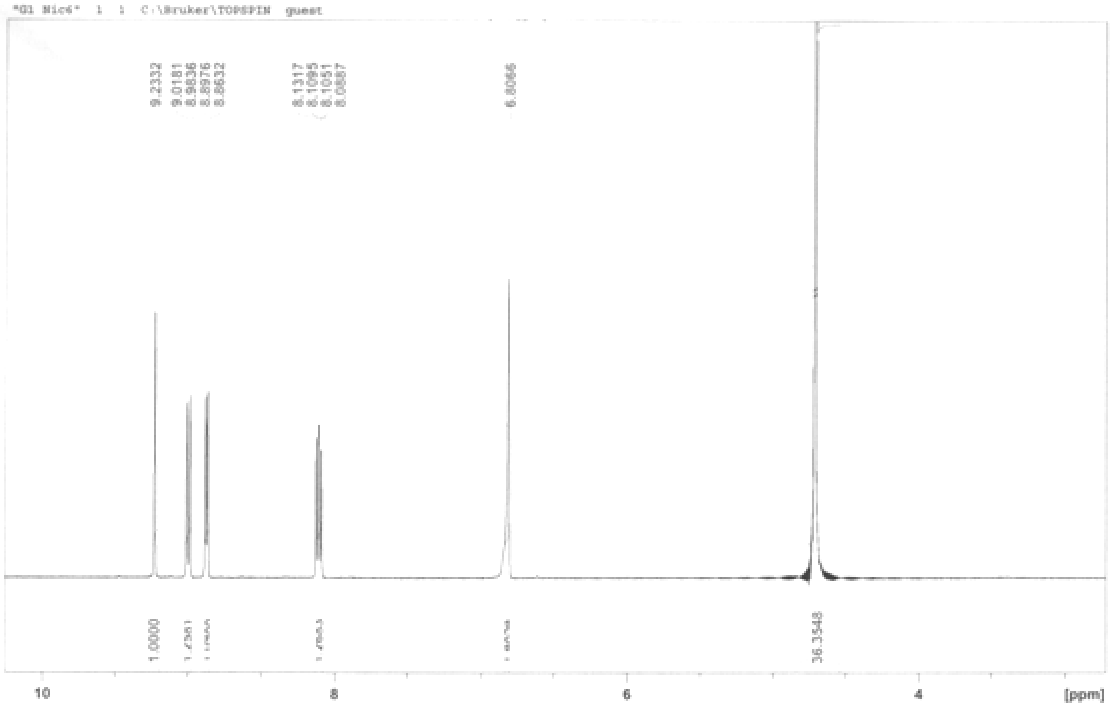

Figure S16. ^{1}H NMR for 5,6,11,12,17,18-hexaazatrinaphthylene-2,3,8,9,14,15-hexanicotamide (**3d**) in DMSO-d_6

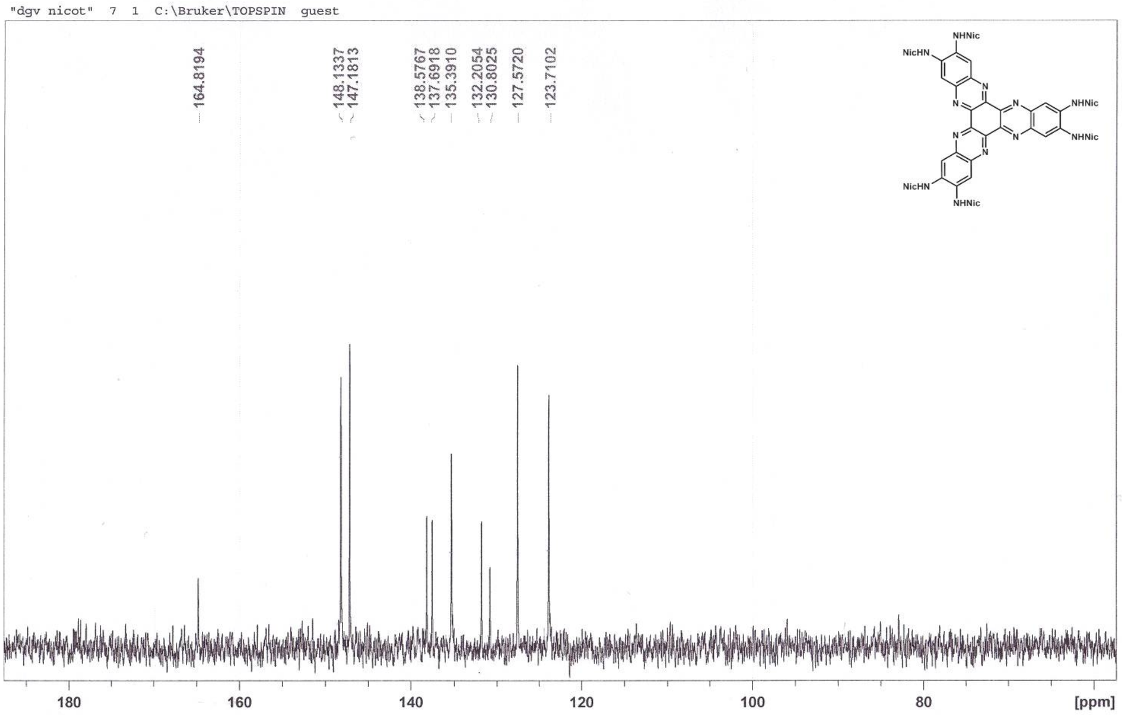

Figure S17. ^{13}C NMR for 5,6,11,12,17,18-hexaazatrinaphthylene-2,3,8,9,14,15-hexanicotamide (**3d**) in DMSO-d_6

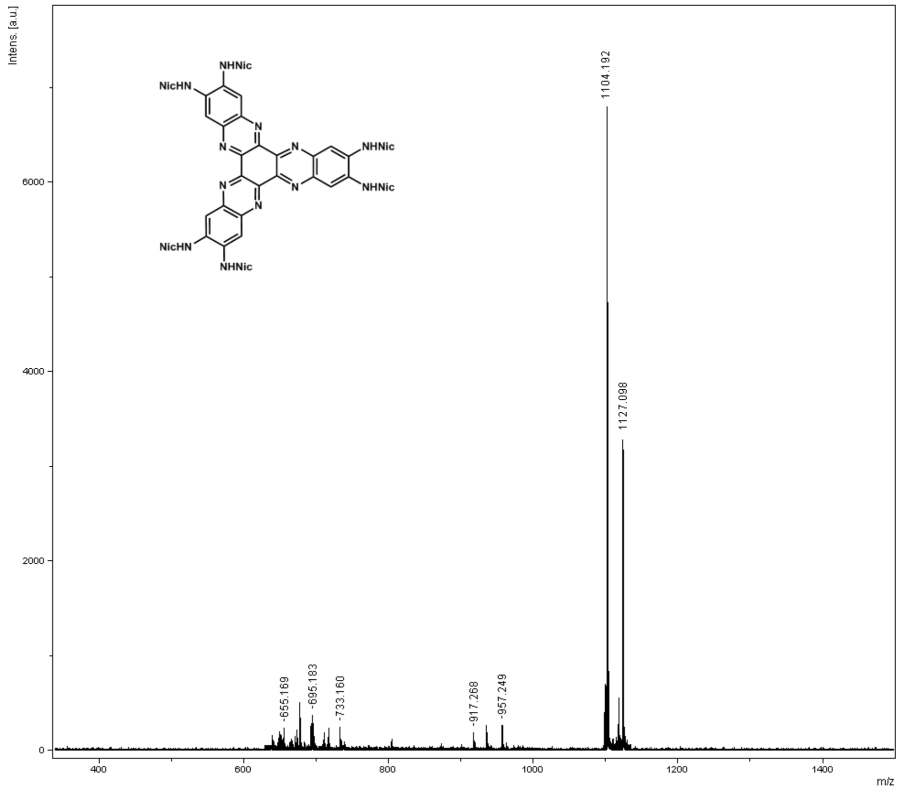

Figure S18. MALDI-TOF for 5,6,11,12,17,18-hexaazatrinaphthylene-2,3,8,9,14,15-hexanicotamide (**3d**)

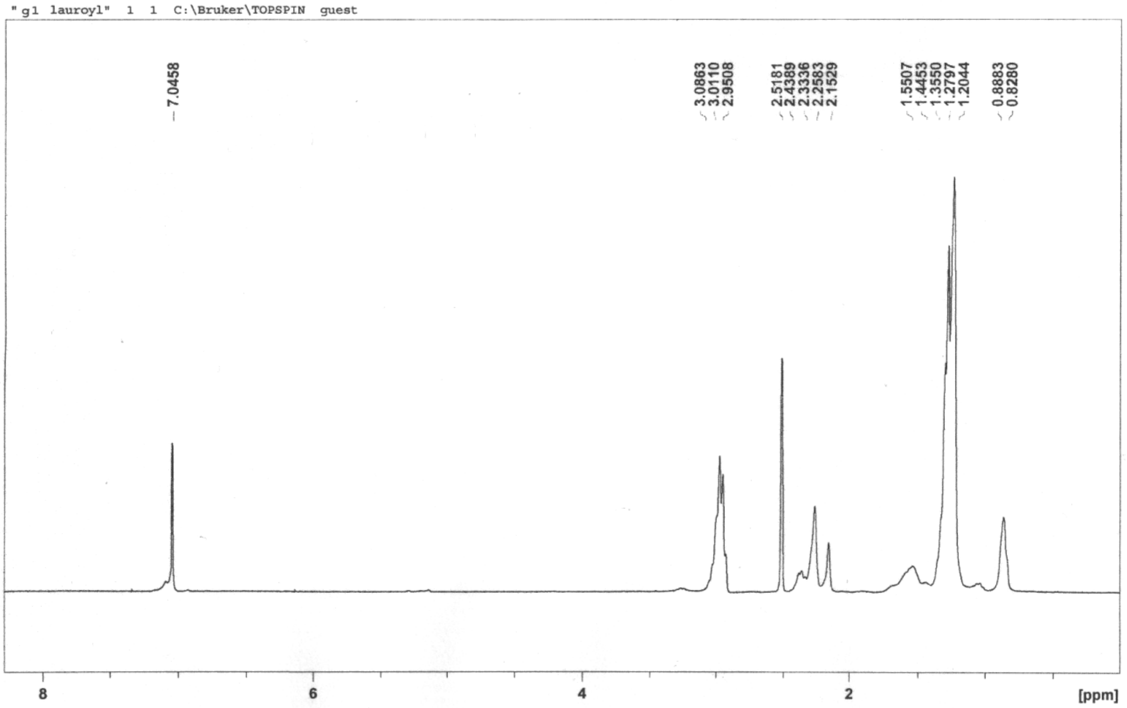

Figure S19. ^{1}H NMR for 5,6,11,12,17,18-hexaazatrinaphthylene-2,3,8,9,14,15-hexadodecanamide (**3b**) in DMSO-d$_6$

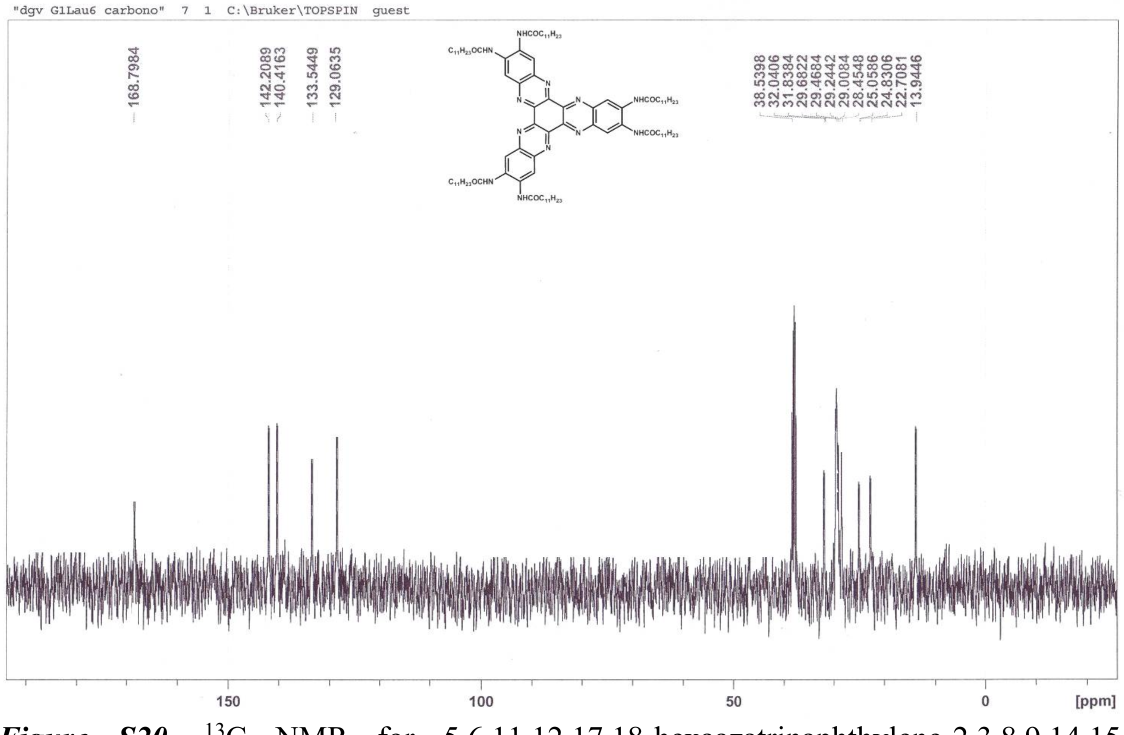

Figure S20. ^{13}C NMR for 5,6,11,12,17,18-hexaazatrinaphthylene-2,3,8,9,14,15-hexadodecanamide (**3b**) in DMSO-d$_6$

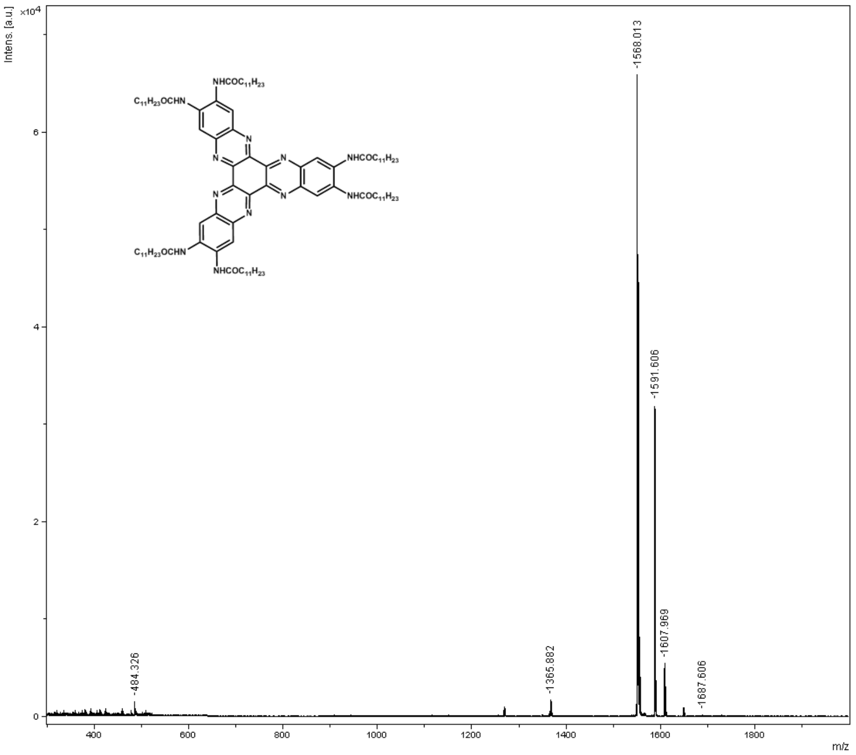

Figure S21. MALDI-TOF for 5,6,11,12,17,18-hexaazatrinaphthylene-2,3,8,9,14,15-hexadodecanamide (**3b**)

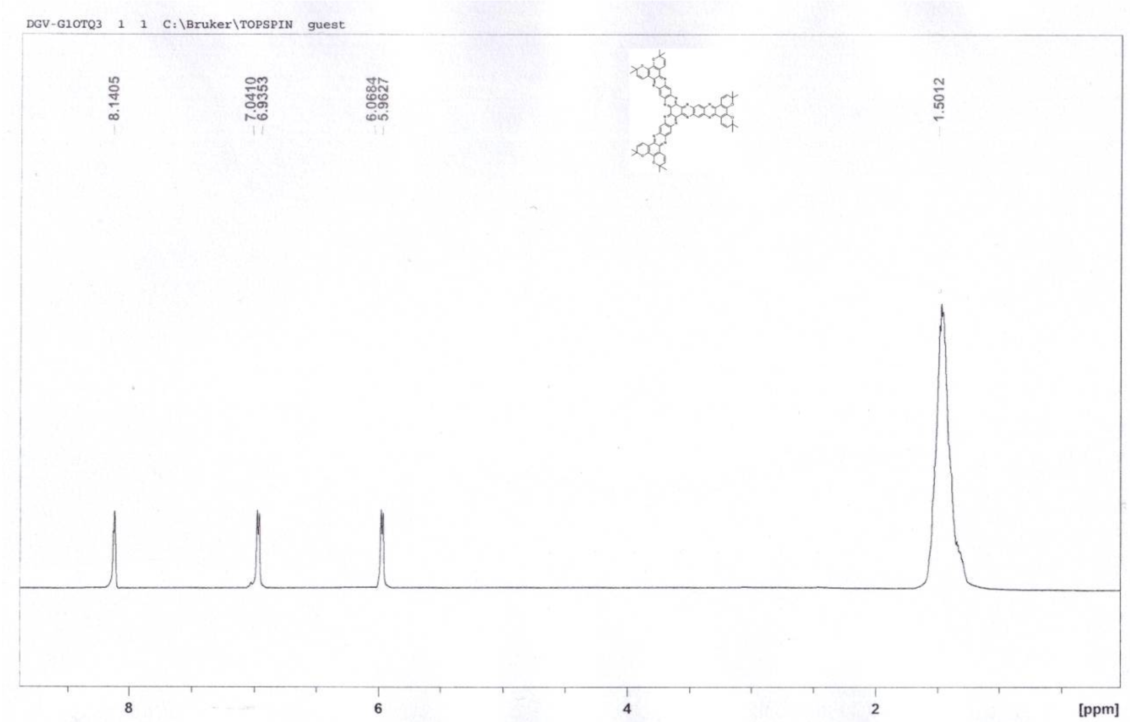

Figure S22. ^{1}H NMR for 5,6,11,12,17,18-hexaazatrinaphthylene-2,3,8,9,14,15-hexaaza-tris(2,2,9,9-tetramethyl-2,5,6,9-tetrahydropyrano[3,2-h]chromene) (**5**) in DMSO-d_6

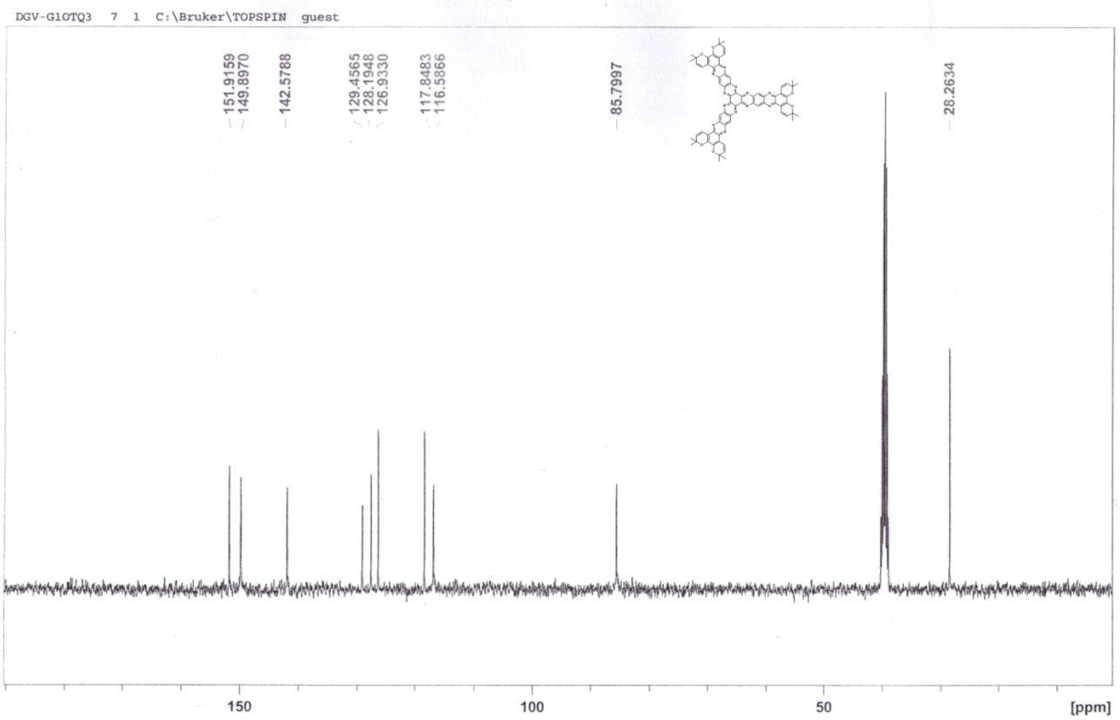

Figure S23. ^{13}C NMR for 5,6,11,12,17,18-hexaazatrinaphthylene-2,3,8,9,14,15-hexaaza-tris(2,2,9,9-tetramethyl-2,5,6,9-tetrahydropyrano[3,2-h]chromene) (**5**) in DMSO-d_6

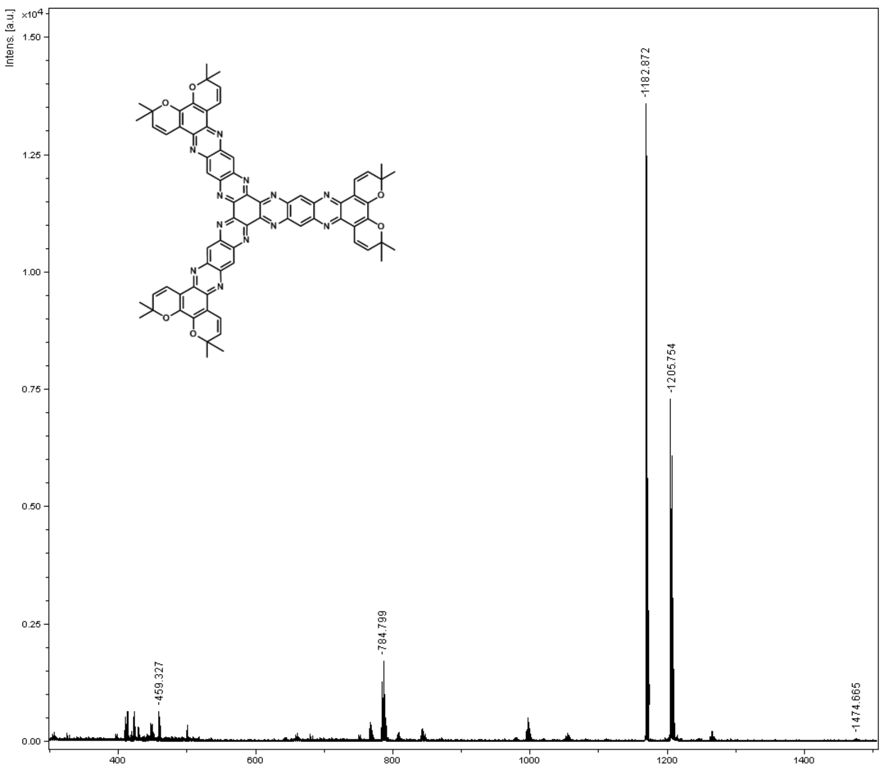

Figure S24. MALDI-TOF for 5,6,11,12,17,18-hexaazatrinaphthylene-2,3,8,9,14,15-hexaaza-tris(2,2,9,9-tetramethyl-2,5,6,9-tetrahydropyrano[3,2-h]chromene) (**5**)

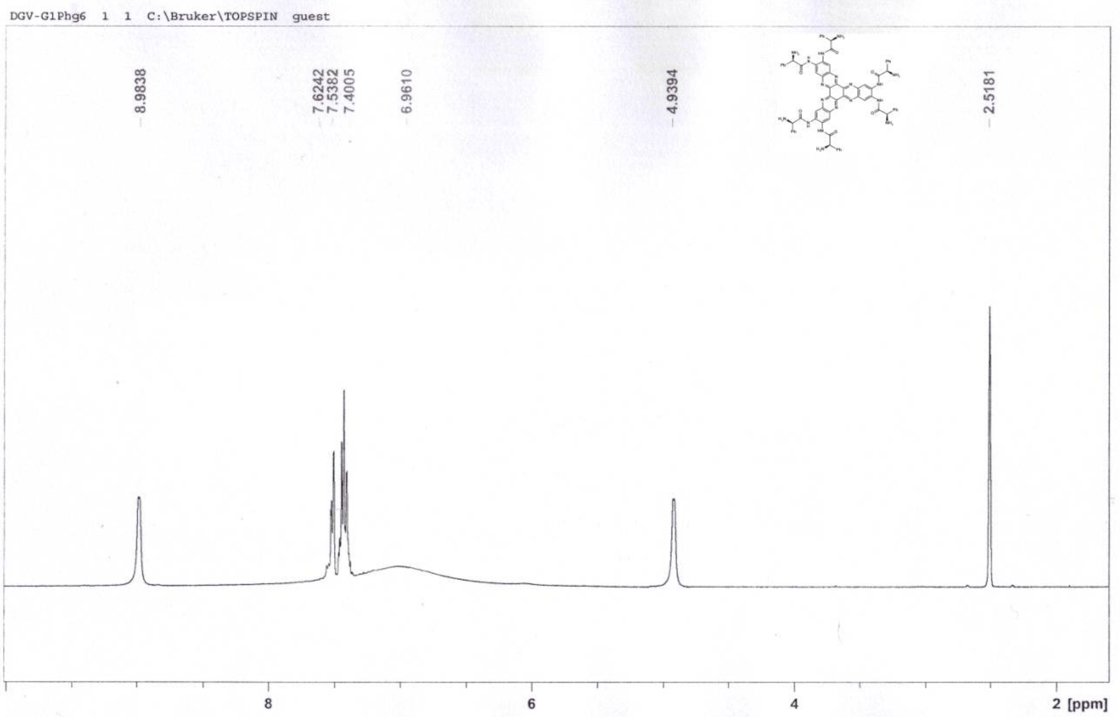

Figure S25. ^{1}H NMR for 5,6,11,12,17,18-hexaazatrinaphthylene-2,3,8,9,14,15-hexa(2R-amino-2R-phenylacetamide) (**3e**) in DMSO-d$_6$

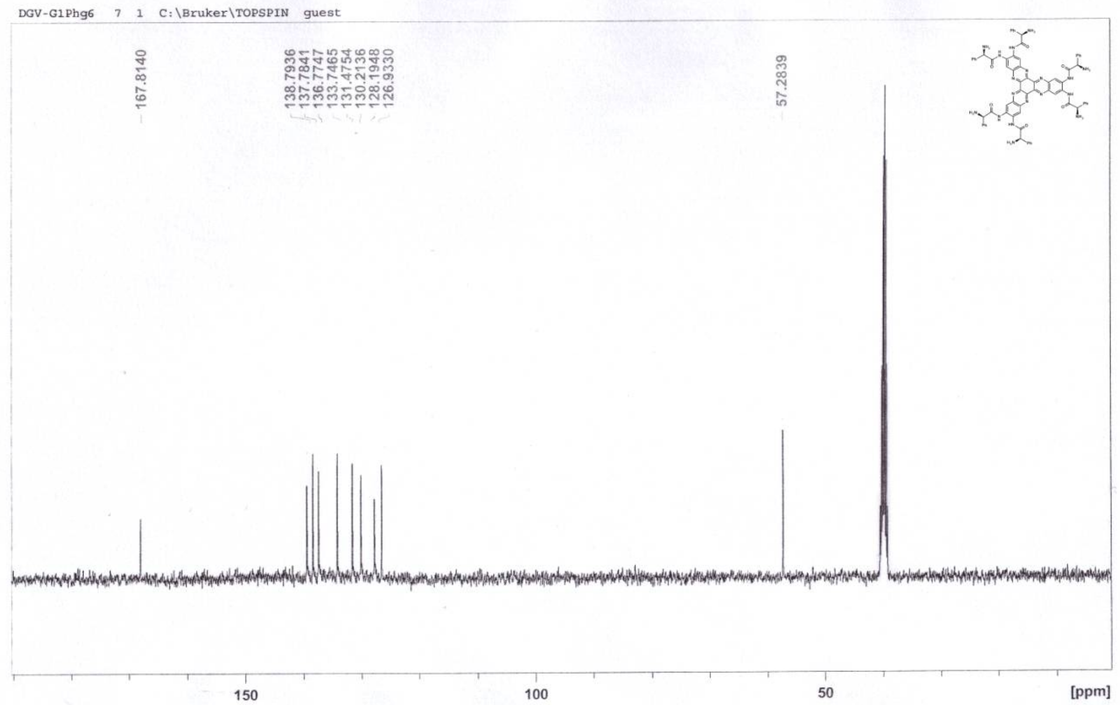

Figure S26. ^{13}C NMR for 5,6,11,12,17,18-hexaazatrinaphthylene-2,3,8,9,14,15-hexa(2R-amino-2R-phenylacetamide) (**3e**) in DMSO-d$_6$

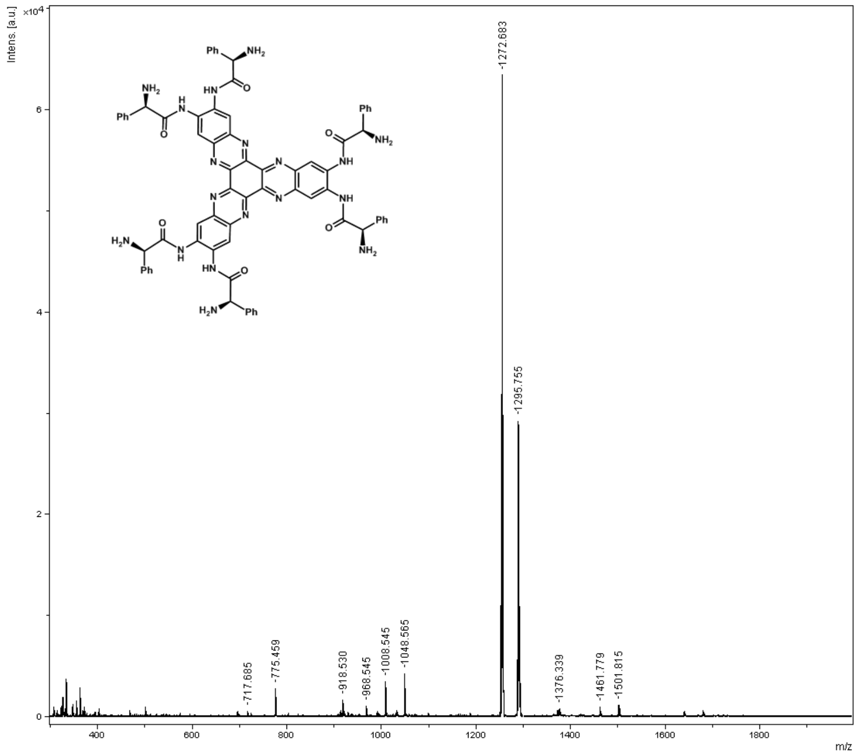

Figure S27. MALDI-TOF for 5,6,11,12,17,18-hexaazatrinaphthylene-2,3,8,9,14,15-hexa(2R-amino-2R-phenylacetamide) (**3e**)

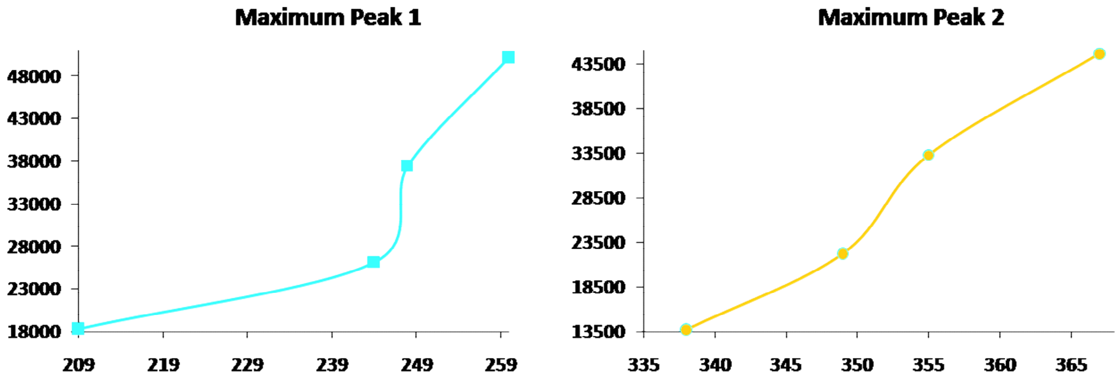

Figure S28. Graphs (ε $M^{-1}cm^{-1}$ *vs.* λ nm) correspond at evolution of two maximum peaks in UV-vis spectra for compound G1 along time. Each point corresponds to days 1, 3, 5 and 7, respectively.

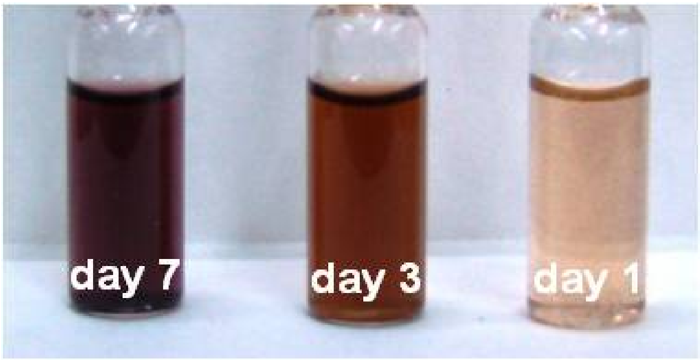

Figure S29. Digital photographs of G1 10^{-5} M in ethanol

4. FT-IR spectra

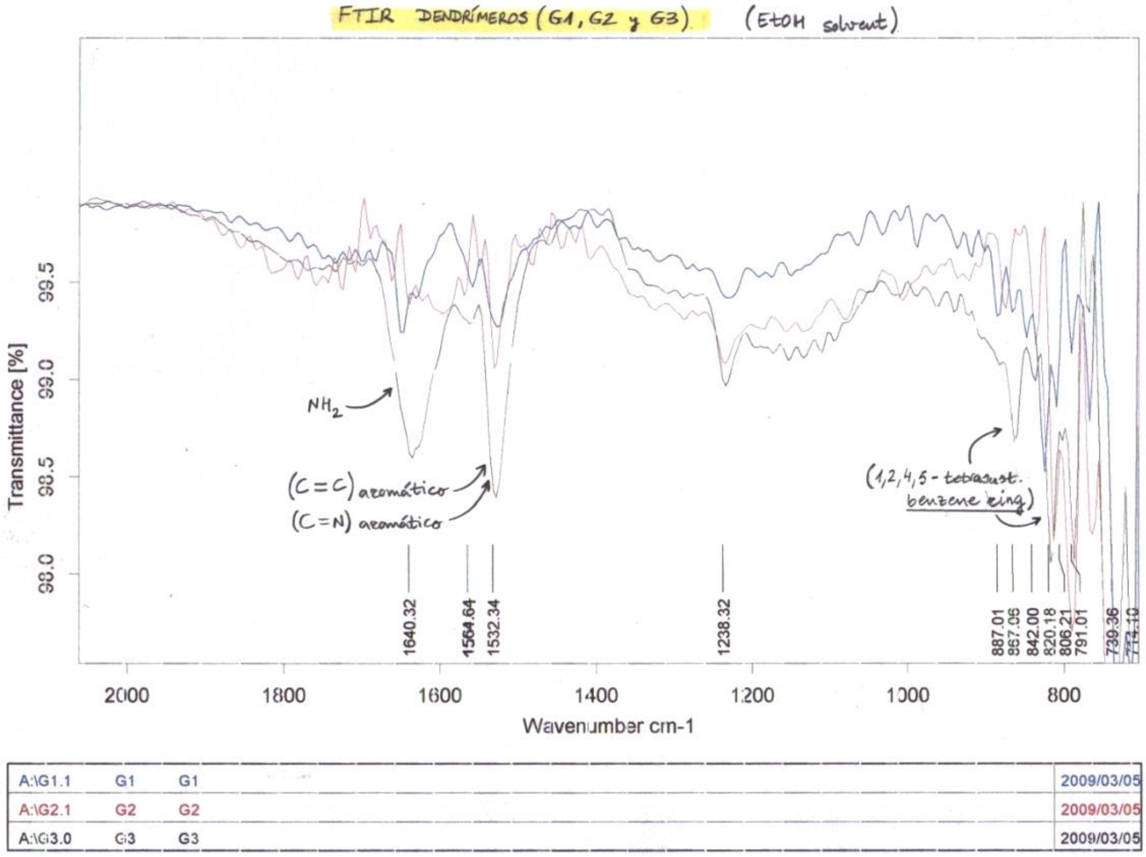

Figure S30. FT-IR for compounds G1, G2 and G3 in ethanol at 20 ºC

FT-IR data in solution state confirmed the presence of amino groups and the 1,2,4,5-tetrasubstituted aromatic ring of compounds **G1**, **G2** and **G3**. Theoretically, up to six hydrogen bonds can be formed between successive coplanar disks within the same column. However, the fractions of intra- and inter- molecular hydrogen bonds were not quantified in the present study. The hydrogen bonds between these compounds should contribute to the induction of the one-dimensional columnar assembly.

The dendritic structures **G1**, **G2** and **G3** present a low solubility in chloroform, dichloromethane and acetone, but are readily soluble in DMF, DMSO, ethanol and water. Therefore, the formation of hydrogen bonds causes the insolubility due to structural defects in columnar ordering. Thus, neighboring columns crosslinking via hydrogen bonding promotes intra-columnar stacking order.[2]

5. Cyclic voltammetry for compound G1

Preliminary studies to evaluate the conductive properties of **G1** have been performed. Cyclic voltammogram of this compound exhibits at least three electrochemical peak couples (AI/CI, AII/CII, AIII/CIII) in the range of +0.75 V to -0.75 V probably related to the formation of the successive radical anions.[3] The anodic wave corresponding to irreversible oxidation (A´I) of **G1** molecule is not observed up to +1.4 V (Fig. S31). This result means that the **G1** HATNA core possesses electron-accepting properties which are in agreement with the electron deficient nature of these compounds.

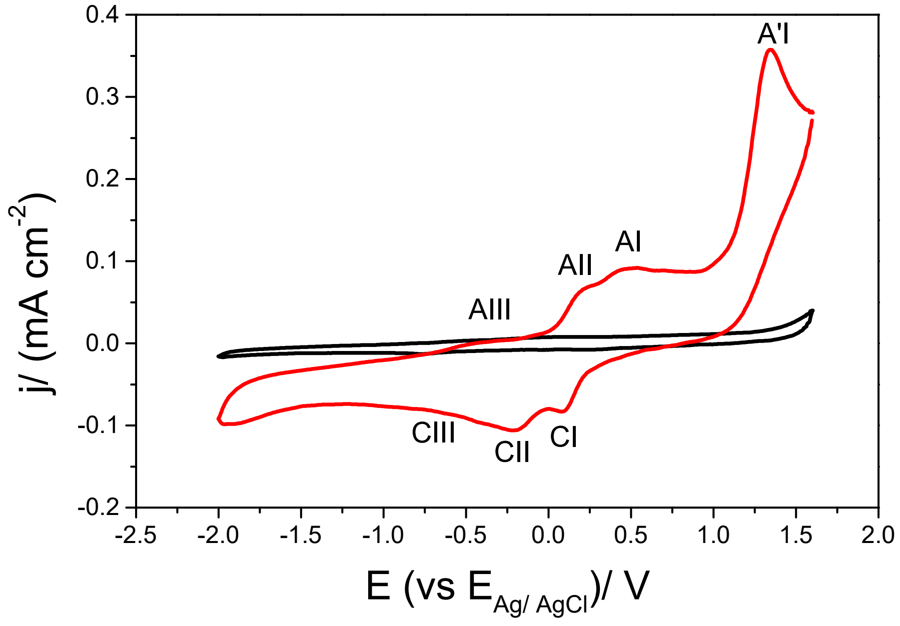

Figure S31. Cyclic voltammogram response recorded at 200 mV s^{-1} for a glassy carbon electrode in DMF/ 0.1 M TBAP, black line, and in G1 10^{-3} M in DMF/ 0.1 M TBAP, red line.

6. Differential Scanning Calorimmetry (DSC) and Thermal Gravimetric Analysis (TGA)

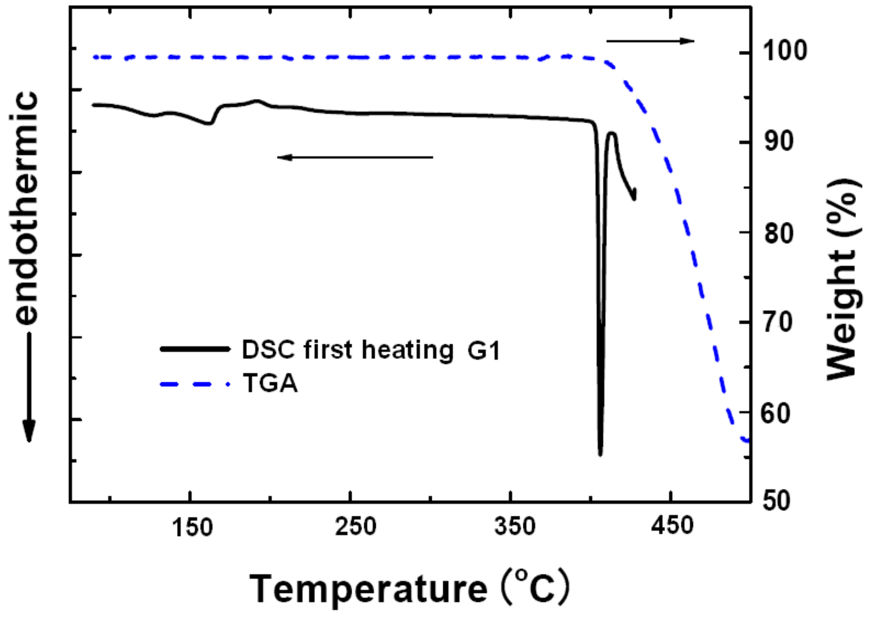

Figure S32. TGA and DSC curve of the first heating for compound **G1**

7. Calculated (DFT B3LYP/6-31G*) 3D structures of compounds

According to molecular modeling, the diameters of **G1** and **G2** are about *ca.* 16.6 and 32.1 nm with a molecular weight of 474 and 1488 u.m.a., respectively (Fig. S33 and S34).

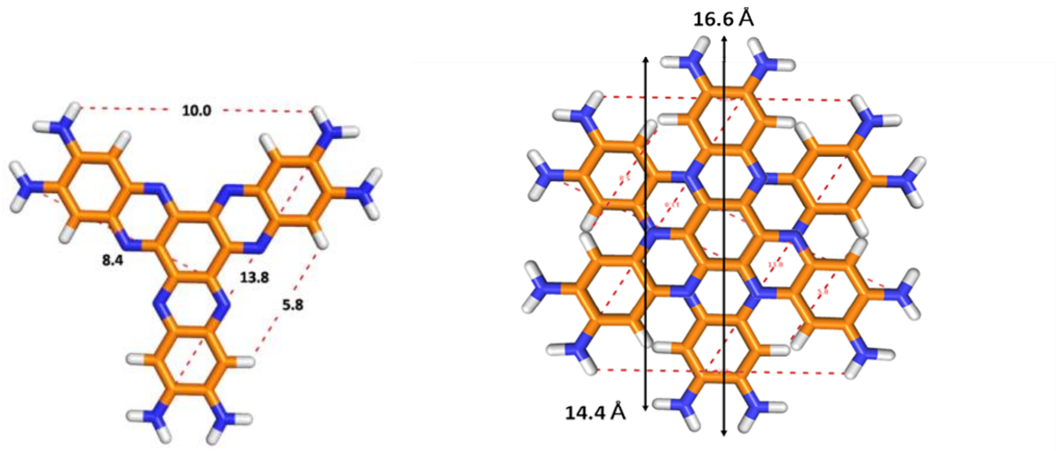

Figure S33. Optimized geometry of **G1** (B3LYP/6-31g*, vacuum) and distances (Å) between atoms (*left*). Representation of two molecules "*face-to-face*" of compound G1 (diameter = *ca*. 16 Å) (*right*)

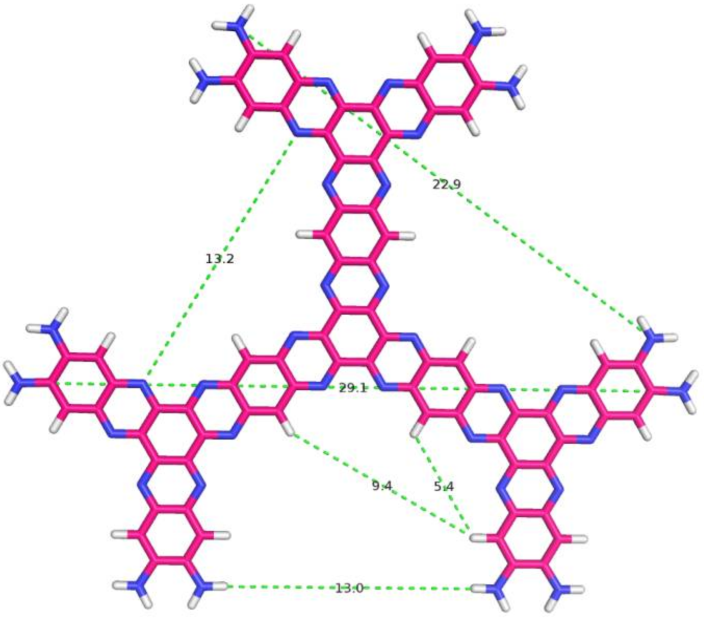

Figure S34. Distances of calculated structure for compound G2

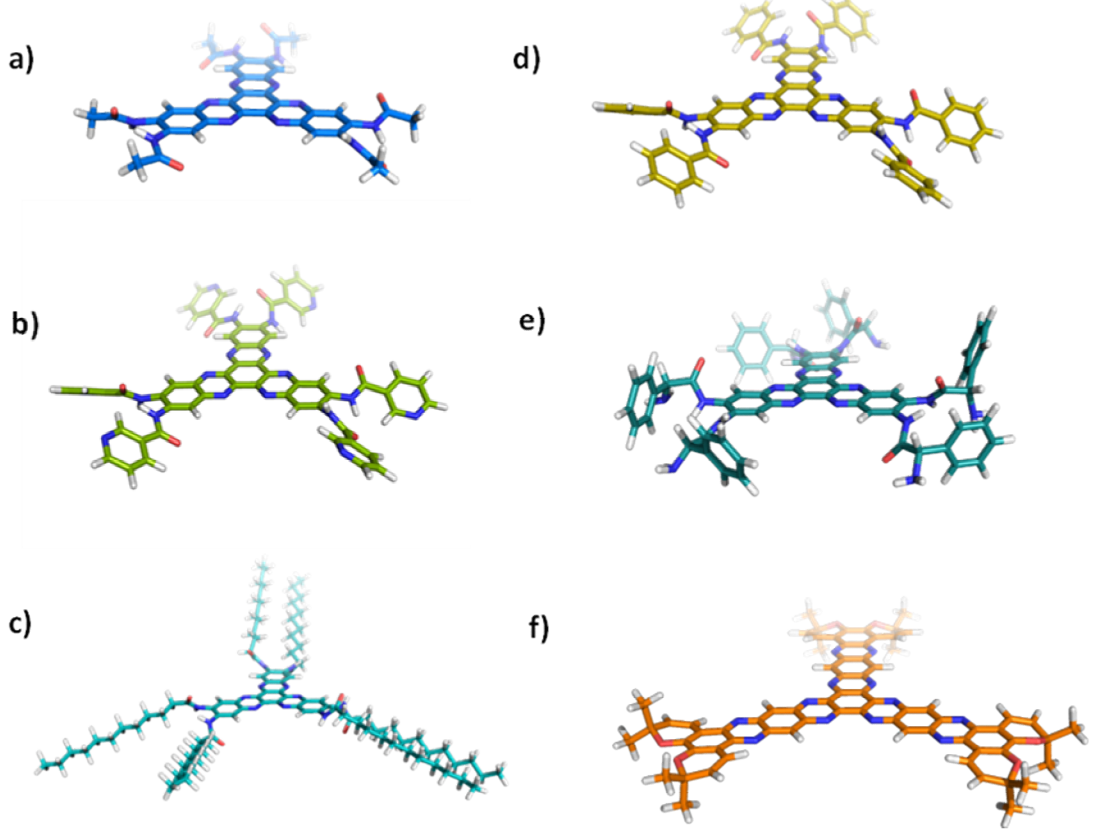

Figure S35. Calculated 3D (B3LYP/6-31G*) structures for compounds **3a** (a), **3d** (b), **3b** (c), **3c** (d), **3e** (e) and **5** (f). The distortion from planarity of the aromatic frameworks of **3a-e** due to the bulky groups brought high solubilities, presumably through the suppression of aggregation of the aromatic π-systems.

8. Mulliken charge distribution

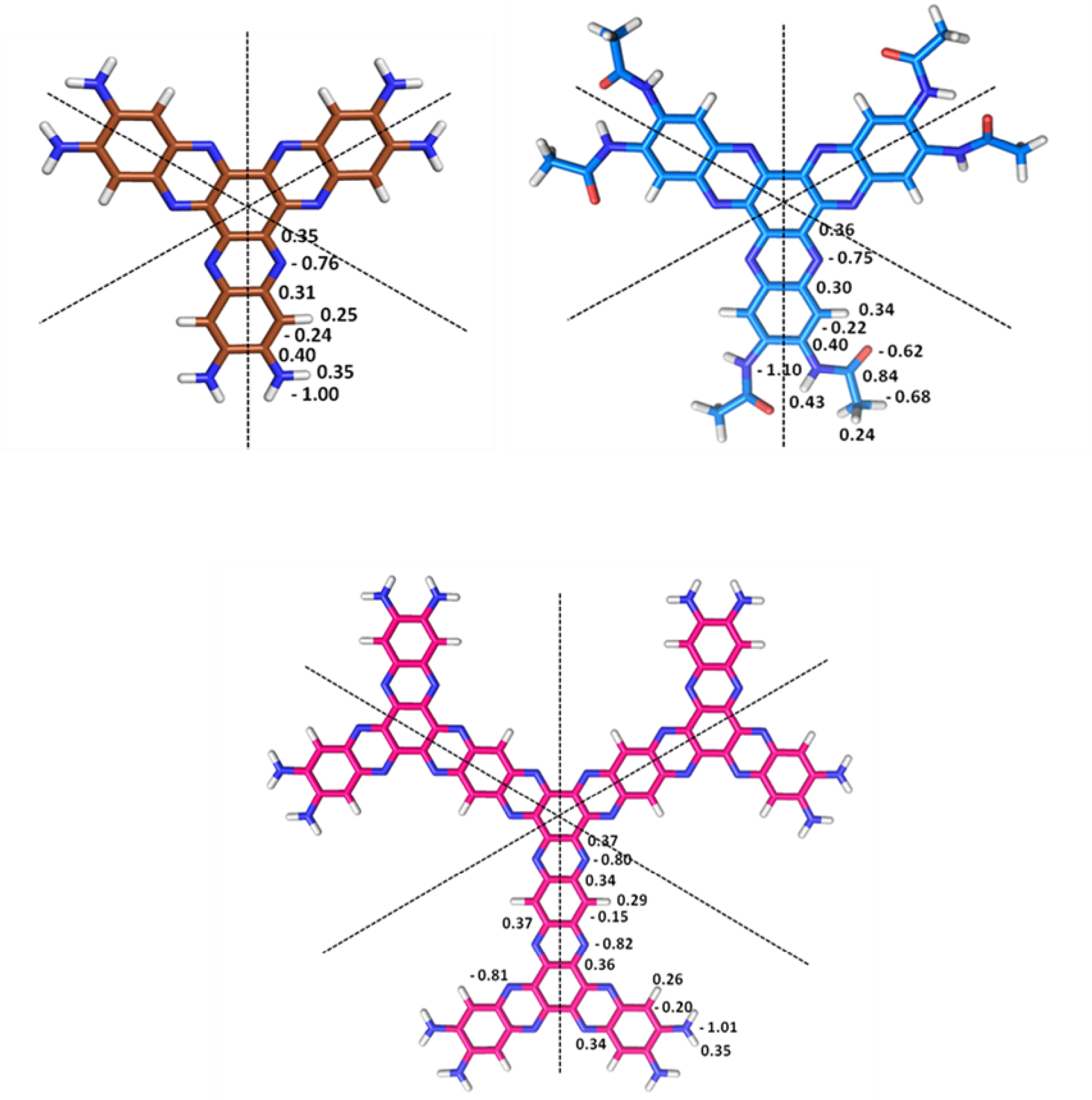

Figure S36. Charge distribution in the compounds G1 (up-left), G2 (down) and 3a (up-right), as calculated at the DFT level from Mulliken population analysis.

9. Schematic drawing of self-assembled molecules

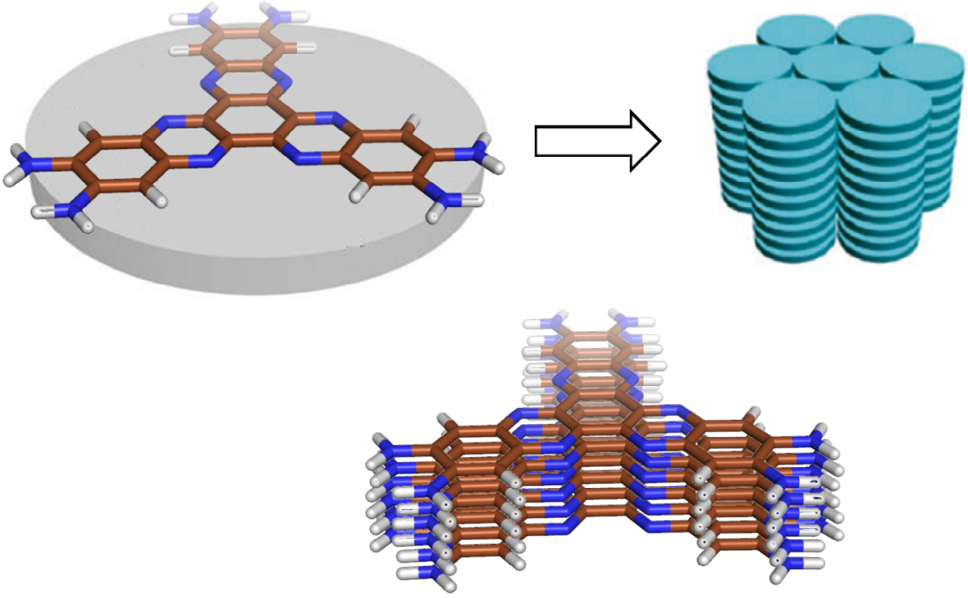

Figure S37. Schematic model of the supramolecular disk assembly of G1 in the ordered columnar phase.

a)

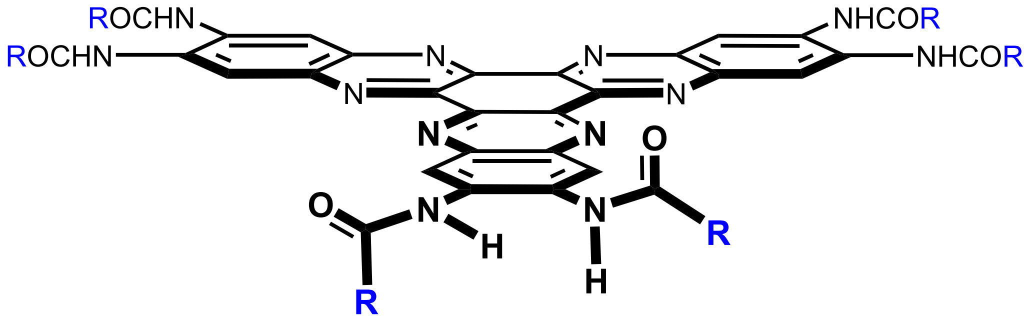

b)

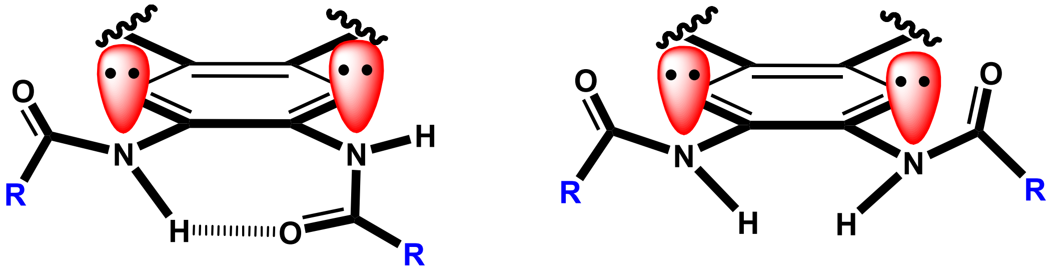

Figure S38. a) Representation of hexaamides 3a-e. b) Formation of intramolecular H-bonding in hexaamides. It is possible that due to the π-conjugation of lone electron pairs of nitrogen atom with the HATNA core, the formation of columnar-aggregate are not allowed by the geometry of the system.

10. Atomic force and Scanning tunneling micrographs

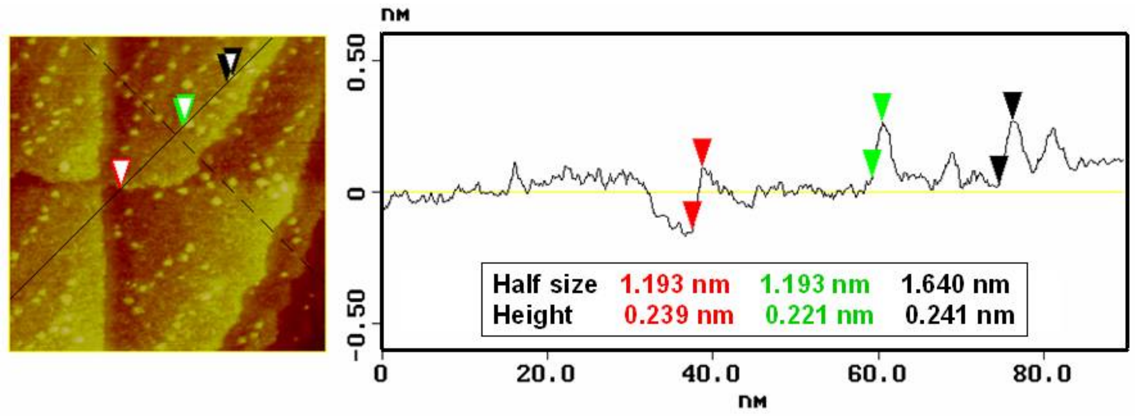

Figure S39. 75nm x 75 nm STM topographical image of the Au(111) surface after 1 min immersion into a G1 10^{-9} M water solution. Representative cross section showing particle dimensions. The red arrows show a mono-atomic step on Au(111) whose height, 0.24 Å, supplies a height reference test.

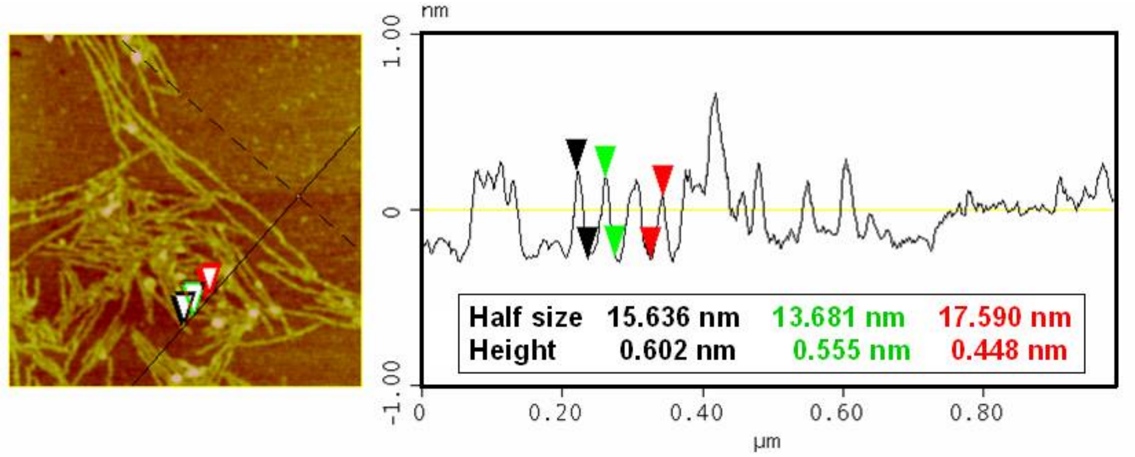

Figure S40. 1.00 µm x 1.00 µm AFM topographical image of the mica surface after 5 min immersion into a G1 10^{-5} M water solution, and a typical cross section showing fibers dimensions.

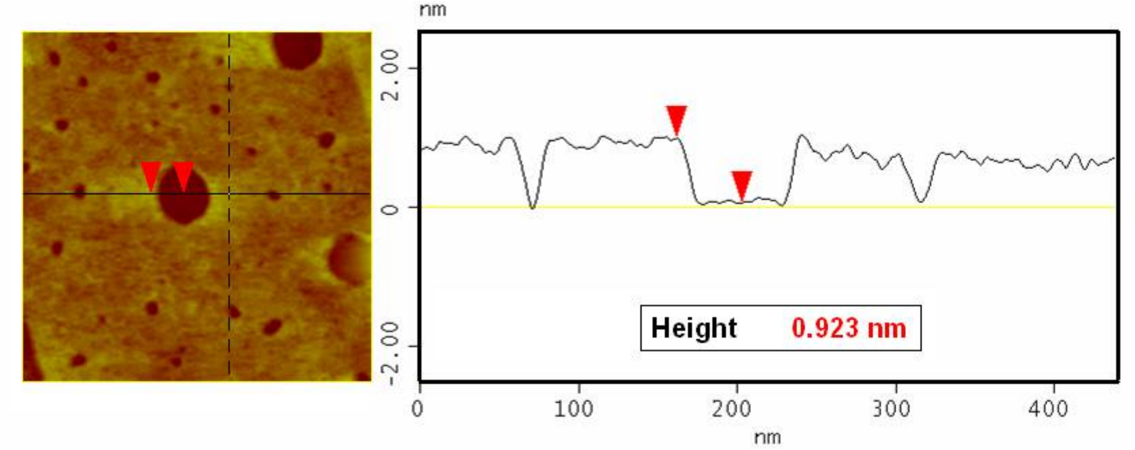

Figure S41. 425nm x 425 nm AFM topographical image of the HOPG surface after 5 min immersion into a G1 10^{-4} M water solution. The film thickness, 0.92 nm, implies that this coverage corresponds to only three G1 molecules (*ca*. 3 Å) thick in a *"face-on"* packing.

11. XRPD

X-ray pattern revealed two main features: a series of reflections at relatively small angles and a reflection at large angles corresponding to Bragg spacing of 0.37 nm (core-core separation), indicating thus a two-dimensional arrangement of the columnar cross-sections in a hexagonal lattice. The latter is a typical feature often observed in columnar mesophases arising due to regular π-π stacking of the discotic mesogens. Presumably, the small angles Bragg reflections in this pattern are oriented along the equator, indicating that the columnar axes are oriented in the meridional direction, perpendicular to the X-ray beam. The side-to-side interaction may be due to lateral hydrogen bonding among free amine units.

X-ray diffraction studies revealed the formation of columnar anisotropic architectures, where the network consists of several discotic stacks packed and the single molecular discs are held together by hydrogen bonds between free NH_2 moieties.

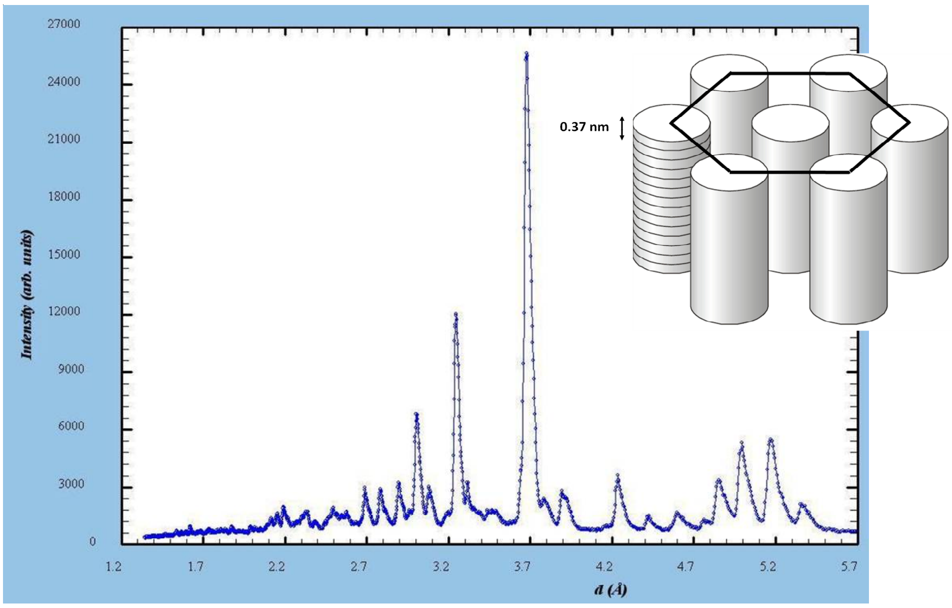

Figure S42. X-ray diffraction pattern of compound **G1** and its one-dimensional intensity *vs.* distance (Å) graph derived from the pattern. Real space representation of the columnar mesophase (*inset*).

12. References y notes

1 G. Kestemont, V. de Halleux, M. Lehmann, D. A. Ivanov, M. Watson and Y. H. Geerts, *Chem. Comm.* 2001, 2074.

2 P. A. Albouy, D. Guillon, B. Heinrich, A.-M. Levelut, J. Malthête, *J. Phys. II* 1995, **5**, 1617.

3 (a) M. Lehmann, G. Kestemont, R. G. Aspe, C. Buess-Herman, M. H. J. Koch, M. G. Debije, J. Piris, M.P. de Haas, J. M. Warman, M.D. Watson, V. Lemaur, J. Cornil, Y.H. Geerts, R. Gearba and D.A. Ivanov, *Chem. Eur. J.* 2005, **11**, 3349. (b) S. Furukawa, T. Okubo, S. Masaoka, D. Tanaka, H-C. Chang, and S. Kitagawa, *Angew. Chem. Int. Ed.* 2005, **44**, 2700-2704. (c) R. Juárez, M.M. Ramos and J.L. Segura, *Tetrah. Lett.* 2007, **48**, 8829.

4 J. Cornil, V. Lemaur, J.P. Calbert and J.L. Brédas, *Adv. Mater.* 2002, **14**, 726.

5 J.H.R. Tucker, M. Gingras, H. Brand and J.-M. Lehn, *J. Chem. Soc., Perkin Trans. 2,* 1997, 1303-1307.
